# Elimination of senescent cells by β-galactosidase-targeted prodrug attenuates inflammation and restores physical function in aged mice

**DOI:** 10.1038/s41422-020-0314-9

**Published:** 2020-04-27

**Authors:** Yusheng Cai, Huanhuan Zhou, Yinhua Zhu, Qi Sun, Yin Ji, Anqi Xue, Yuting Wang, Wenhan Chen, Xiaojie Yu, Longteng Wang, Han Chen, Cheng Li, Tuoping Luo, Hongkui Deng

**Affiliations:** 1 The MOE Key Laboratory of Cell Proliferation and Differentiation, College of Life Sciences, Peking-Tsinghua Center for Life Sciences, and School of Basic Medical Sciences, State Key Laboratory of Natural and Biomimetic Drugs, Peking University Health Science Center, Peking University, Beijing, 100191 China; 2 State Key Laboratory of Chemical Oncogenomics, School of Chemical Biology & Biotechnology, Peking University Shenzhen Graduate School, Shenzhen, Guangdong 518055 China; 3 Peking-Tsinghua Center for Life Sciences, Academy for Advanced Interdisciplinary Studies, Peking University, Beijing, 100871 China; 4 Key Laboratory of Bioorganic Chemistry and Molecular Engineering, Ministry of Education and Beijing National Laboratory for Molecular Science, College of Chemistry and Molecular Engineering, Peking University, Beijing, 100871 China; 5 School of Life Sciences, Joint Graduate Program of Peking-Tsinghua-NIBS, Peking University, Beijing, 100871 China; 6 School of Life Sciences, Center for Bioinformatics, Center for Statistical Science, Peking University, Beijing, 100871 China

**Keywords:** Senescence, Apoptosis

## Abstract

Cellular senescence, a persistent state of cell cycle arrest, accumulates in aged organisms, contributes to tissue dysfunction, and drives age-related phenotypes. The clearance of senescent cells is expected to decrease chronic, low-grade inflammation and improve tissue repair capacity, thus attenuating the decline of physical function in aged organisms. However, selective and effective clearance of senescent cells of different cell types has proven challenging. Herein, we developed a prodrug strategy to design a new compound based on the increased activity of lysosomal β-galactosidase (β-gal), a primary characteristic of senescent cells. Our prodrug SSK1 is specifically activated by β-gal and eliminates mouse and human senescent cells independently of senescence inducers and cell types. In aged mice, our compound effectively cleared senescent cells in different tissues, decreased the senescence- and age-associated gene signatures, attenuated low-grade local and systemic inflammation, and restored physical function. Our results demonstrate that lysosomal β-gal can be effectively leveraged to selectively eliminate senescent cells, providing a novel strategy to develop anti-aging interventions.

## Introduction

Aging is the predominant risk for physiological degeneration, increased chronic morbidities, and age-specific mortality.^1,2^ One major hallmark of aging is the chronic accumulation of cellular senescence, a permanent state of cell-cycle arrest in response to various damaging stimuli.^3,4^ Cellular senescence impairs the ability of tissue regeneration and drives chronic low-grade inflammation, which exacerbates the aging process.^5,6^ Importantly, transplantation of senescent cells into young mice was sufficient to drive age-related pathology and cause persistent physical dysfunction.^7^ In contrast, deletion of senescent cells by a genetic approach attenuated age-related deterioration and extended the health-span in aged mice.^8,9^ These studies demonstrated that senescence is one of the major drivers of aging and that clearing senescent cells is a promising approach to treat age-related diseases and improve physical function.^6,10^

Previous studies have shown that compounds termed ‘senolytics’ could kill senescent cells.^11–13^ Reported senolytics target anti-apoptotic pathways, which are up-regulated to inhibit apoptosis in senescent cells.^11,14^ These senolytics have been reported to eliminate certain types of senescent cells and have shown the potential to improve physiological function in several tissues.^7,12,15^ However, senolytic drugs have significant limitations in killing senescent cells in terms of specificity and broad-spectrum activity because of the dynamic and highly heterogeneous nature of the senescence program, which leads to the varying sensitivity of different types of senescent cells to current senolytic drugs.^6,16,17^ To overcome these challenges, it is highly demanded to develop a new strategy that permits selectively deleting senescent cells in a wide spectrum of cell types or tissues for anti-aging interventions.

To specifically target senescent cells, we focused on one primary characteristic of senescent cells — the increased activity of lysosomal β-galactosidase, exploited as senescence-associated β-galactosidase (SA-β-gal).^18,19^ Notably, SA-β-gal in diverse types of senescent cells is one widely used marker for identifying senescence in vitro and in vivo, which is linked to the increased content of lysosomes.^20–23^ Therefore, we hypothesized that lysosomal β-gal could be utilized for the design of a galactose-modified prodrug to target senescent cells in a broader spectrum. This prodrug could be processed into a cytotoxic compound by β-gal and subsequently delete senescent cells in a specific manner, a strategy that could overcome the limitations of current senolytic drugs.

Here, we designed a new prodrug that was specifically cleaved by lysosomal β-gal into cytotoxic gemcitabine and induced apoptosis in senescent cells. This prodrug eliminated both mouse and human senescent cells independent of the senescence inducers and cell types. In aged mice, our compound reduced SA-β-gal-positive senescent cells in different tissues, decreased senescence- and age-associated gene signatures, attenuated low-grade chronic inflammation, and improved physical function.

## Results

### Design of SSK1 to kill senescent cells selectively

**Fig. 1:**
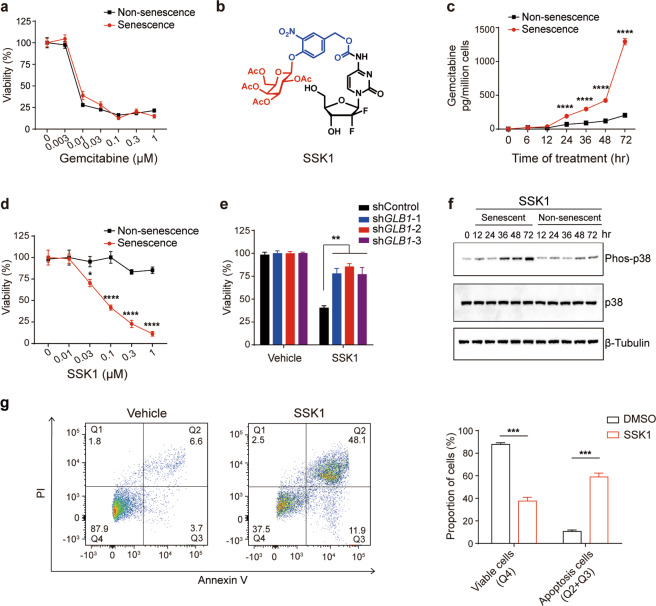
Design of SSK1 and validation of its ability to selectively kill senescent cells. **a** Quantification of cell viability of non-senescent and replication-induced senescent new born dermal fibroblasts (NBFs) incubated with increasing concentrations of gemcitabine for 3 days (*n* = 3). **b** Molecular structure of the prodrug SSK1. **c** Metabolism of SSK1 into gemcitabine in replication-induced senescent NBFs and their non-senescent counterparts incubated with SSK1 (0.5 µM) for 3 days (*n* = 3). **d** Quantification of cell viability of senescent and non-senescent NBFs incubated with increasing concentrations of SSK1 for 3 days (*n* = 4). **e** Cell viability of *GLB1* knockdown (sh*GLB1*-1, -2 and -3) or shControl senescent cells treated with vehicle or SSK1 (0.5 µM) for 3 days (*n* = 4). For cell viability analysis in (**a**), (**d**) and (**e**), cell numbers were quantified using Hoechst 33342 staining and dead cells were excluded by propidium iodide (PI) staining.** f** Detection of phos-p38 MAPK in senescent cells or non-senescent cells incubated with SSK1 (0.5 µM) for 3 days by western blot. **g** Representative flow cytometric plots (left) and quantification (right) of the percentage of viable (Q4: PI^−^ annexin V^−^) and apoptotic (Q2 and Q3: PI^+^ annexin V^+^ and PI^−^ annexin V^+^) cells in vehicle- or SSK1-treated senescent cells by annexin V and PI staining after vehicle or SSK1 (0.5 µM) treatment for 3 days (*n* = 3). ‘*n*’ represents the number of biological replicates. Data are presented as means ± SEM. Unpaired two-tailed *t-*test for (**c**), (**d**), and (**g**), two-way ANOVA test for (**e**), *P < 0.05, ***P < 0.001, ****P < 0.0001.

To design a lysosomal β-gal-responsive prodrug, we first screened a panel of FDA-approved drugs to select a compound with potent cytotoxicity for senescent cells as an end-product (Supplementary information, Table S1). We chose gemcitabine because (1) it killed both mouse and human senescent cells effectively and potently (Fig. 1a; Supplementary information, Fig. S1a–c and Table S1); (2) its 4-amino group is a well-established site for prodrug development;^24^ and (3) it exhibited reduced systemic toxicity due to its short plasma circulation time.^25,26^ Therefore, we synthesized senescence-specific killing compound 1 (SSK1) (Fig. 1b), in which the acetyl group and β-gal-responsive moiety improved the cellular permeability and specificity, respectively.^27^ Gemcitabine has been reported to be transported into cells via molecular transporters for nucleosides given its hydrophilicity,^24,25^ however SSK1 was modified with acetyl group and β-gal-responsive moiety suggesting its hydrophobicity (Fig. 1b). Nucleoside transporter inhibitor was unable to block the killing effect of SSK1 indicating that SSK1 entered the senescent cells independent of transporter (Supplementary information, Fig. S1e). We also validated that the prodrug SSK1 was specifically cleaved to release cytotoxic gemcitabine in senescent cells but not in non-senescent cells (Fig. 1c; Supplementary information, Fig. S1d). These results suggested that SSK1 was toxic to senescent cells and non-toxic to non-senescent cells.

To test SSK1’s ability to selectively kill senescent cells, we treated primary mouse fibroblasts and their replication-induced senescent counterparts with SSK1. We found that SSK1 selectively and potently eliminated β-gal-positive senescent cells within a wide therapeutic window (Fig. 1d; Supplementary information, Fig. S1f–i). Since SSK1 was cleaved into 4-(hydroxymethyl)-2-nitrophenol and gemcitabine in senescent cells, as a control, the hydrolyzed product of SSK1 — 4-(hydroxymethyl)-2-nitrophenol showed no obvious toxicity to senescent and non-senescent cells (Supplementary information, Fig. S1j), and gemcitabine killed both cells at the similar concentration (Fig. 1a). To address whether SSK1 cleared senescent cells through β-gal rather than the higher metabolic rates in senescent cells, we tested another gemcitabine-based prodrug (LY2334737) lacking the galactose moiety and found no obvious difference in the elimination of senescent cells and non-senescent cells (Supplementary information, Fig. S1k). We further used RNA interference to decrease the expression of *GLB1*, the gene encoding β-gal (Supplementary information, Fig. S1l).^28^ Knockdown of *GLB1* reduced SA-β-gal activity (Supplementary information, Fig. S1m) and showed little effect on other senescence markers, such as *p16*, *p21* and *Il1α* (Supplementary information, Fig. S1n). More importantly, knockdown of *GLB1* impaired the ability of SSK1 to kill SA-β-gal-positive senescent cells (Fig. 1e), suggesting that its specificity for senescent cells depended on lysosomal β-gal activity. Collectively, we leveraged lysosomal β-gal, one conserved characteristic of senescent cells to design a prodrug that specifically killed senescent cells.

Next, we explored the molecular mechanism of SSK1 in senescent cells. As gemcitabine has been reported to induce cell death through the activation of p38 mitogen-activated protein kinase (MAPK),^29,30^ we examined the phosphorylation status of p38 MAPK and its upstream MKK3/MKK6 in SSK1-treated senescent cells by western blot.^31,32^ After SSK1 treatment, both p38 MAPK and MKK3/MKK6 were activated by phosphorylation in senescent cells (Fig. 1f; Supplementary information, Fig. S2a, b), indicating that SSK1 could be processed into gemcitabine in senescent cells and activated the p38 MAPK signaling pathway. This was further confirmed by the treatment of p38 MAPK inhibitors Birb796, SB203580, and SB202190, which impaired SSK1’s ability to specifically kill senescent cells (Supplementary information, Fig. S2c). Thus, SSK1 killed senescent cells through the activation of the p38 MAPK signaling pathway. We also found that SSK1 was able to induce mitochondrial DNA damage in senescent cells (Supplementary information, Fig. S2d), similar to the reported ganciclovir, which also belongs to the nucleoside analogs as gemcitabine.^33^ Additionally, flow cytometry analysis showed that SSK1 induced senescent cells into annexin V and propidium iodide double-positive cells, and western blot result showed SSK1 could activate caspase 3, which indicated that SSK1 killed senescent cells by inducing apoptosis (Fig. 1g; Supplementary information, Fig. S2b). These results suggested that our prodrug SSK1 was activated by lysosomal β-gal and selectively killed senescent cells through the activation of p38 MAPK and induction of apoptosis.

### SSK1 kills senescent cells in a broader manner

**Fig. 2:**
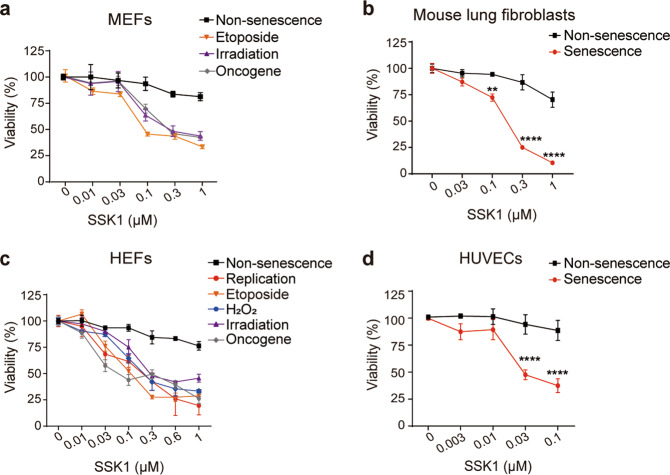
SSK1 kills senescent cells in a broader manner. **a** Quantification of cell viability of non-senescent and senescent mouse embryonic fibroblasts (MEFs) induced by etoposide, irradiation (10 Gy) or oncogene (*Kras*^*G12V*^) incubated with increasing concentrations of SSK1 for 3 days (*n* = 3). Significance analysis is shown in Supplementary information, Table S2. **b** Quantification of cell viability of non-senescent mouse lung fibroblasts from 3-month-old mice and replication-induced senescent counterparts incubated with increasing concentrations of SSK1 for 3 days (*n* = 4). **c** Quantification of cell viability of non-senescent and senescent human embryonic fibroblasts (HEFs) induced by replication, etoposide, H_2_O_2_ (200 µM), irradiation (10 Gy) or oncogene (*Kras*^*G12V*^) incubated with SSK1 for 3 days (*n* = 3). Significance analysis is shown in Supplementary information, Table S2. **d** Quantification of cell viability of non-senescent and replication-induced senescent HUVECs incubated with increasing concentrations of SSK1 for 3 days (*n* = 4). ‘*n*’ represents the number of biological replicates. Data are presented as means ± SEM. Unpaired two-tailed *t-*test for (**b**) and (**d**), two-way ANOVA test for (**a**) and (**c**), **P < 0.01, ****P < 0.0001.

We then tested the specificity of SSK1 for mouse and human senescent cells. First, we used SSK1 to treat mouse embryonic fibroblasts (MEFs) in which senescence was induced by ionizing radiation, oncogene (*Kras*^*G12V*^) overexpression, or genotoxic stress (etoposide treatment). These senescent cells induced by various stimuli were selectively killed by SSK1, while non-senescent cells were largely unaffected (Fig. 2a; Supplementary information, Table S2). Second, we found that SSK1 could selectively kill replication-induced senescent lung fibroblasts from adult mice compared with non-senescent counterparts (Fig. 2b). Third, to understand if there is any efficacy difference of SSK1 between species, we treated senescent human embryonic fibroblasts (HEFs) induced by different stimuli with SSK1. The results showed that SSK1 selectively cleared human senescent cells induced by different stresses in a dose-dependent manner, without an obvious effect on non-senescent counterparts (Fig. 2c; Supplementary information, Fig. S2e, f and Table S2). We also treated human umbilical vein endothelial cells (HUVECs) and human preadipocytes with SSK1, and found that senescent cells were selectively killed by SSK1 when compared with their non-senescent counterparts (Fig. 2d; Supplementary information, Figs. S2g–i and S3e). Collectively, our results showed that SSK1 selectively killed both mouse and human senescent cells in a cell type- and senescence stimulus-independent manner.

We further compared the specificity of SSK1 with other three reported senolytic drugs (ABT263, dasatinib plus quercetin, and fisetin) on HEFs, human preadipocytes and HUVECs, which were previously used to study senolytics.^11,12,17^ In our study, SSK1 selectively killed senescent HEFs induced by different stimuli (Supplementary information, Fig. S3a and Table S2). ABT263 could also specifically kill senescent HEFs induced by different stresses but in a narrow concentration range (Supplementary information, Fig. S3b and Table S2). On the other hand, the combination of dasatinib and quercetin (D + Q) showed limited selectivity as it killed both senescent and non-senescent HEFs (Supplementary information, Fig. S3c and Table S2), and fisetin only killed certain senescent HEFs at high concentration (Supplementary information, Fig. S3d and Table S2). SSK1 also specifically killed senescent human preadipocytes (Supplementary information, Fig. S3e), while ABT263 and fisetin had limited elimination effect on this cell type (Supplementary information, Fig. S3f, h). D + Q could kill senescent human preadipocytes at high concentration with an affection of non-senescent cells (Supplementary information, Fig. S3g). Additionally, the experiments on HUVECs showed that SSK1 selectively eliminated senescent cells with a broader concentration spectrum compared with ABT263 (Supplementary information, Fig. S3i, j), whereas D + Q showed no selectivity to senescent and non-senescent cells (Supplementary information, Fig. S3k). These results demonstrated that SSK1 has higher specificity and potency to eliminate senescent cells.

### SSK1 decreases senescent cells in lung-injured mice

**Fig. 3:**
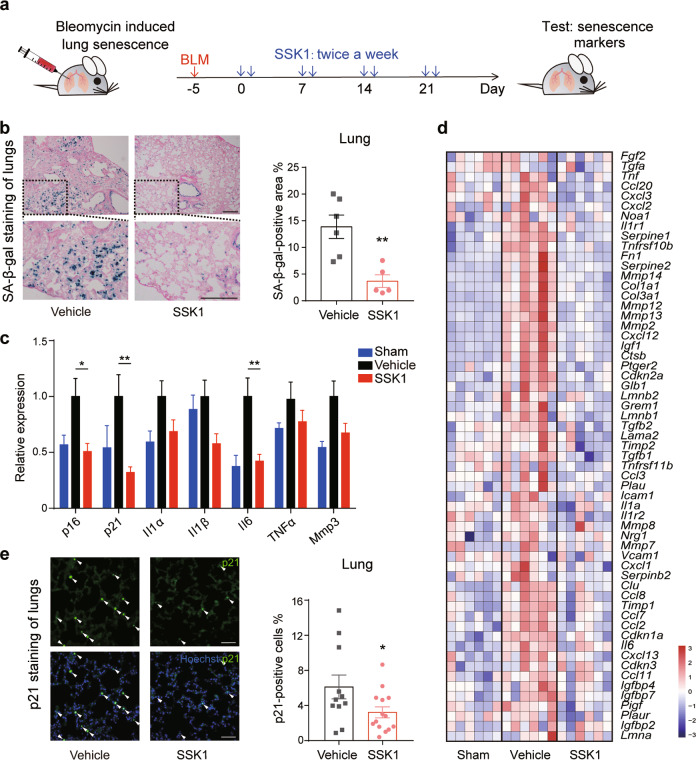
SSK1 eliminates SA-β-gal-positive senescent cells in a bleomycin-induced lung injury model. **a** Experimental design for bleomycin-induced lung injury. Mice (3–6-month-old) were subjected to transtracheal injection of bleomycin (1.5 mg/kg) or saline (sham surgery, Sham) 5 days before drug treatment. Lung-injured mice were intraperitoneally injected with SSK1 (0.5 mg/kg) or vehicle (DMSO) consecutive two days every week for four weeks; sham surgery mice were treated with DMSO in the same way. **b** Representative images (left) and quantification (right) of SA-β-gal staining of lungs from bleomycin-injured mice after vehicle or SSK1 treatment (vehicle-treated, *n* = 6; SSK1-treated, *n* = 5). Scale bars, 200 µm. **c** Expression of senescence-associated genes by RT-qPCR in lungs of sham surgery and bleomycin-injured mice treated with vehicle or SSK1 (Sham, *n* = 9; vehicle-treated, *n* = 9; SSK1-treated, *n* = 10). **d** Heatmap of senescence-related gene expression of lungs from sham surgery and bleomycin-injured mice treated with vehicle or SSK1 (Sham, *n* = 6; vehicle-treated, *n* = 6; SSK1-treated, *n* = 6). **e** Representative images (left) and quantification (right) of p21 immunofluorescence of lungs from bleomycin-injured mice after vehicle or SSK1 treatment (vehicle-treated, *n* = 11; SSK1-treated, *n* = 14). White arrowheads show p21-positive cells. Scale bars, 50 µm. Each data point represents an individual mouse. ‘*n’* represents the number of mice. Data are presented as means ± SEM. Unpaired two-tailed *t-*test, *P < 0.05, **P < 0.01.

Next, we examined the effect of SSK1 on senescent cells in vivo. We employed two independent in vivo models of senescence: stress-induced senescence and naturally occurring senescence. First, we induced DNA strand break and cellular senescence in mouse lungs by intratracheal instillation of bleomycin.^34,35^ SA-β-gal-positive senescent cells were easily induced in these mice with lung injury (Supplementary information, Fig. S4a, b), a commonly used model to study elimination of senescent cells.^36–38^ We treated these lung- injured mice with vehicle or SSK1 by intraperitoneal injection (Fig. 3a) and found that SSK1 significantly reduced the percentage of SA-β-gal-positive cells in lung by 3.8-fold compared with that in vehicle-treated lung-injured mice (Fig. 3b). RT-qPCR results revealed that SSK1 also decreased senescence-associated markers compared with vehicle treatment (Fig. 3c). In consistent with this result, our RNA-seq results indicated that the expression of senescence-related genes was significantly decreased in lung tissues from SSK1-treated group (Fig. 3d). We further confirmed that SSK1 reduced p21-positive cells by immunofluorescence staining (Fig. 3e). Taken together, our results indicated that SSK1 could eliminate SA-β-gal-positive cells and decrease senescence-associated markers in vivo.

We further found that lung fibrosis and the expression of fibrosis genes, including *Col1a1*, *Col3a1* and *Fn1*, an outcome mediated by the accumulation of senescence,^36,39^ were reduced by SSK1 treatment (Fig. 3d; Supplementary information, Fig. S4c, d). We then evaluated the change of systemic health parameters following SSK1 treatment. We found that the treatment of SSK1 was beneficial to the body weight recovery in bleomycin-induced lung-injured mice (Supplementary information, Fig. S4e). Especially, exercise capacity and muscle strength measured by treadmill and grip strength respectively were significantly increased in SSK1-treated mice compared with vehicle-treated mice (Supplementary information, Fig. S4f, g). Additionally, SSK1 treatment showed a tendency to increase the survival rate (Supplementary information, Fig. S4h). These results suggested that SSK1-induced reduction of SA-β-gal-positive senescent cells provides a potential method to alleviate senescence-related lung injury.

### SSK1 attenuates the senescence-associated secretory phenotype (SASP) and age-associated signatures in aged mice

**Fig. 4:**
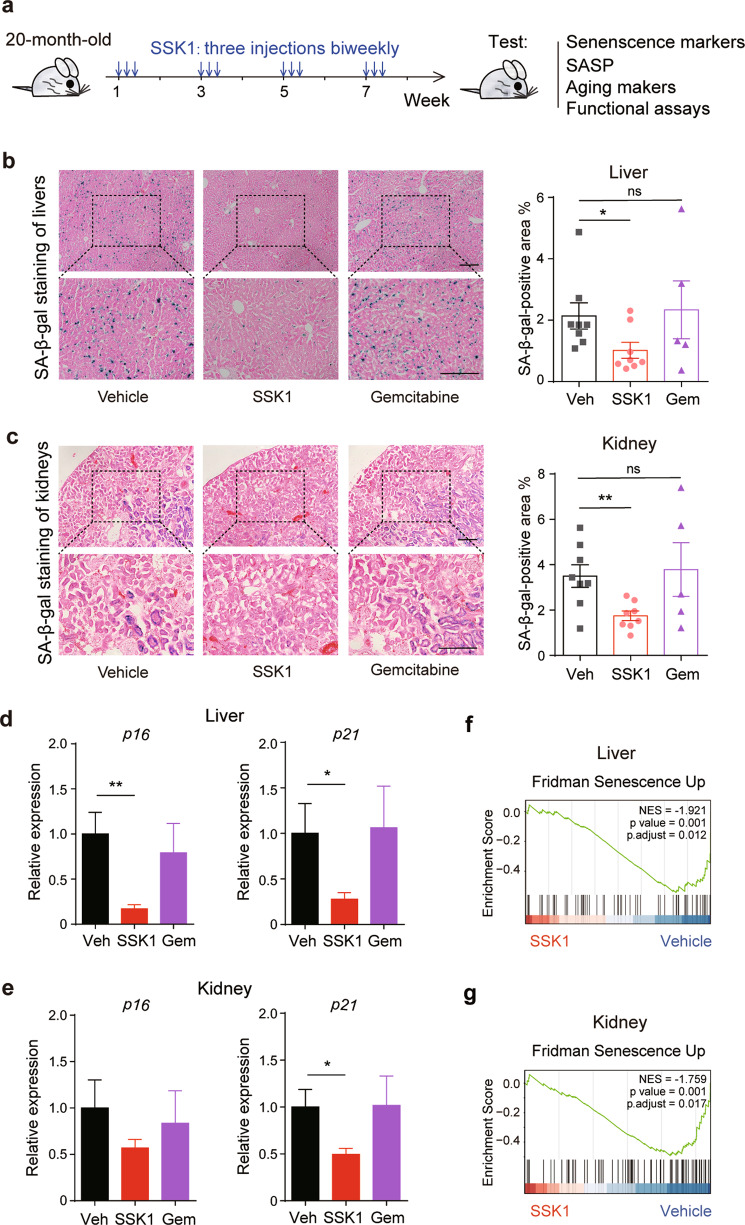
SSK1 deletes senescent cells and attenuates senescence-associated signatures in aged mice. **a** Experimental design for SSK1 treatment of aged mice. Old mice (20–22-month-old) were intraperitoneally injected with SSK1 (0.5 mg/kg), gemcitabine (0.5 mg/kg) or vehicle (DMSO) for continued 3 days every 2 weeks for 8 weeks. **b**, **c** Representative images (left) and quantification (right) of SA-β-gal staining of livers (**b**) and kidneys (**c**) from old mice treated with vehicle (Veh), SSK1 or gemcitabine (vehicle-treated, *n* = 8; SSK1-treated, *n* = 8; gemcitabine-treated, *n* = 5). Scale bars, 200 µm. **d**, **e** Expression of *p16* and *p21* analyzed by RT-qPCR in livers (**d**) and kidneys (**e**) from mice treated with vehicle, SSK1 or gemcitabine. For (**d**): vehicle-treated, *n* = 6; SSK1-treated, *n* = 8; gemcitabine-treated, *n* = 5. For (**e**): vehicle-treated, *n* = 8; SSK1-treated, *n* = 8; gemcitabine-treated, *n* = 5. **f**, **g** GSEA blots of the statistically significant gene set: Fridman Senescence Up in livers (**f**) and kidneys (**g**) from old mice treated with vehicle or SSK1. For (**f**): vehicle-treated, *n* = 5; SSK1-treated, *n* = 4. For (**g**): vehicle-treated, *n* = 5; SSK1-treated, *n* = 5. Each data point represents an individual mouse. ‘*n*’ represents the number of mice. Data are presented as means ± SEM. Unpaired two-tailed *t-*test, *P < 0.05, **P < 0.01.

To further examine the effect of SSK1 on naturally occurring senescent cells in aged mice, we treated 20-month-old C57BL/6 mice with SSK1, gemcitabine or vehicle intermittently for 8 weeks (Fig. 4a). Senescent cells accumulated in different tissues of aged mice,^4,40,41^ which was consistent with our observation that SA-β-gal-positive cells increased in aged livers, kidneys and lungs (Supplementary information, Fig. S5a–c). We found that SSK1 reduced SA-β-gal-positive cells in liver, kidney and lung tissues of aged mice compared with vehicle and gemcitabine (Fig. 4b, c; Supplementary information, Fig. S5d). SSK1 treatment also decreased the expression of other senescence markers, including the CDK inhibitors *p16* and *p21* in aged mice as indicated by RT-qPCR analysis compared with vehicle and gemcitabine treatment (Fig. 4d, e). Additionally, SSK1 treatment in aged mice could down-regulate the gene signatures associated with senescence as shown by gene set enrichment analysis (GSEA) in both livers and kidneys (Fig. 4f, g). These results indicated that SSK1 reduced naturally accumulated senescent cells and decreased senescence markers in mice.

**Fig. 5:**
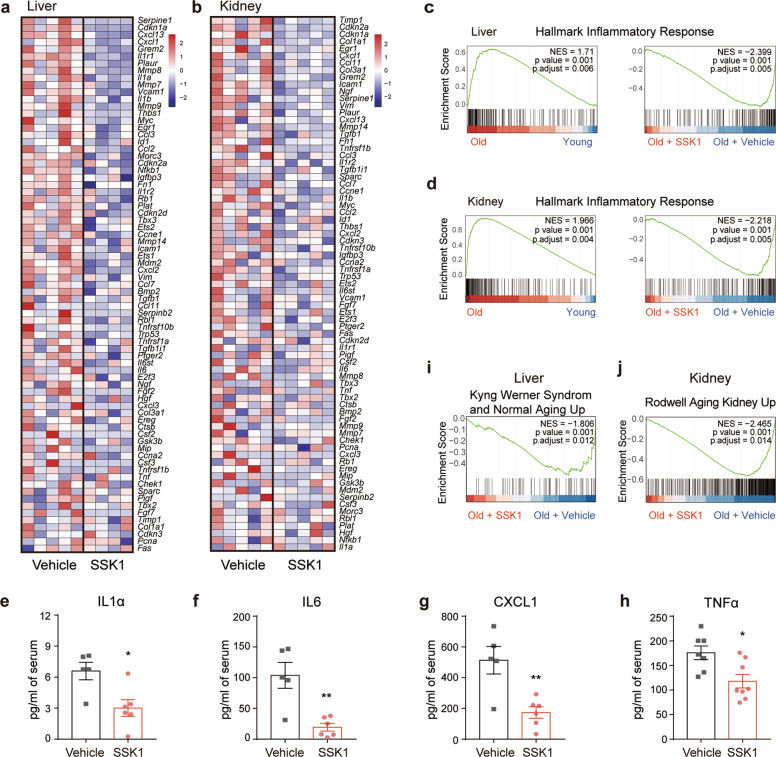
SSK1 attenuates the SASP and aging-associated signatures in aged mice. **a**, **b** Heatmaps of senescence-related gene expression of livers (**a**) and kidneys (**b**) treated with vehicle or SSK1 (0.5 mg/kg). **c**, **d** GSEA results of the statistically significant gene set: Hallmark Inflammatory Response. Left: Gene sets enriched in the livers (**c**) and kidneys (**d**) from old mice compared with young mice. Right: Gene sets down-regulated in the livers (**c**) and kidneys (**d**) of old mice treated with SSK1 compared with vehicle. **e**–**h** IL1α (**e**), IL6 (**f**), CXCL1 (**g**), and TNFα (**h**) protein levels in blood serum from old mice treated with vehicle or SSK1 measured by ELISA. **i** GSEA of a statistically significant gene set: Kyng Werner Syndyom and Normal Aging Up of livers down-regulated in the old mice treated with SSK1 compared with vehicle. **j** GSEA of a statistically significant gene set: Rodwell Aging Kidney Up of kidneys down-regulated in the old mice treated with SSK1 compared with vehicle. For (**a**), (**c**), and (**i**): vehicle-treated, *n* = 5; SSK1-treated, *n* = 4. For (**b**), (**d**), and (**j**): vehicle-treated, *n* = 5; SSK1-treated, *n* = 5. For (**e**–**g**): vehicle-treated, *n* = 5, SSK1-treated, *n* = 6. For (**h**): vehicle-treated, *n* = 7; SSK1-treated, *n* = 8. Each data point represents an individual mouse. ‘*n*’ represents the number of mice. Data are presented as means ± SEM. Unpaired two-tailed *t-*test, *P < 0.05, **P < 0.01.

Senescent cells secrete a range of inflammatory cytokines, chemokines, proteinases, and other factors, collectively called the SASP.^5,42^ SASP contributes to local and systemic low-grade inflammation during aging, to age-related degenerative phenotypes, and to impairment of physical function.^5,6,43^ We found that SSK1 attenuated the expression pattern of SASP-associated genes in aged livers and kidneys by RNA sequencing (Fig. 5a, b). Additionally, GSEA analysis showed that SSK1 down-regulated genes associated with inflammatory response, the TNFα/NF-κB signaling pathway, the IL6/JAK/STAT3 signaling axis and the complement system trending toward the level of their young counterparts (Fig. 5c, d; Supplementary information, Fig. S6a–f). Similar results were also shown by gene ontology (GO) enrichment analysis (Supplementary information, Fig. S7a–d and Table S3). We also measured several key factors of SASP in serum from aged mice and observed the decrease of IL1α, IL6, CXCL1, and TNFα from the SSK1-treated group compared with the vehicle-treated group (Fig. 5e–h).

In addition to senescent cells, *macrophages* are also reported to cause age-associated chronic inflammation.^44,45^ Since the accumulated *macrophages* tend to display senescence features such as the increased activity of SA-β-gal and high expression level of *p16*,^44,46^ we also studied the effect of SSK1 on *macrophages*. We performed immunofluorescence analysis of F4/80-positive *macrophages*, and found that these cells accumulated in livers of aging mice and that SSK1 could attenuate the macrophage infiltration (Supplementary information, Fig. S6g). This could partially account for the decreased inflammatory cytokines in aged mice. The decline of macrophage accumulation was further confirmed by our RNA-seq data of livers analyzed by xCell (Supplementary information, Fig. S6h, i).^47^ Notably, these results were consistent with our findings in the lung injury model, in which a reduction of β-gal-positive *macrophages* was observed in lungs of the SSK1-treatd mice with a decrease of inflammatory factors (Supplementary information, Fig. S6j). Collectively, these results showed that SSK1 efficiently alleviated SASP both locally and systemically, which were probably due to elimination of accumulated senescent cells and *macrophages* in aged organisms.

We also tested the effect of SSK1 on age-associated signatures by GSEA analysis. The treatment of SSK1 decreased the age-associated signatures in aged livers and kidneys (Fig. 5i, j), suggesting a potential beneficial effect on the clearance of SA-β-gal-positive senescent cells in aged tissues. We further performed GO analysis and found that some pathways which were closely related to liver function (oxidation-reduction process, metabolic process, fatty acid metabolic process, and mitochondrion) were up-regulated in SSK1-treated aged livers (Supplementary information, Fig. S7f and Table S3). On the contrary, these GO pathways were down-regulated in aged livers compared with young livers (Supplementary information, Fig. S7e and Table S3). Additionally, we found that SSK1 could attenuate liver fibrosis as revealed by Masson fibrosis staining (Supplementary information, Fig. S7g). These findings suggested that the clearance of senescent cells by SSK1 was beneficial to alleviate the aging signatures in aged mice.

### SSK1 improves the physical function of aged mice

**Fig. 6:**
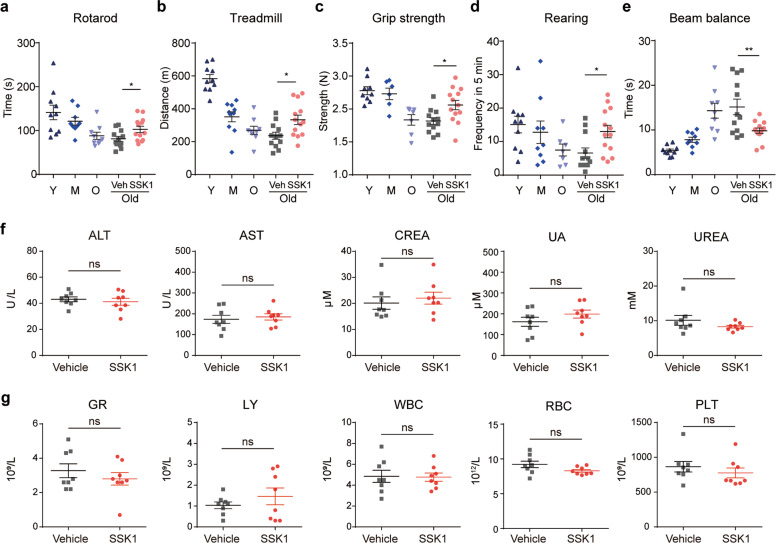
SSK1 improves physical function of aged mice. **a** Quantification of maximal time in rotarod of old mice treated with vehicle (Veh) or SSK1 (0.5 mg/kg) compared with young (Y), middle-aged (M), old (O) mice control (*n* = 12, 13, 10, 9, 9 for each group, respectively). **b** Exhaustion distance in treadmill of old mice treated with vehicle or SSK1 compared with young, middle-aged, old mice control (*n* = 12, 13, 10, 10, 9 for each group, respectively). **c** Grip strength of four limbs of old mice treated with vehicle or SSK1 compared with young, middle-aged, old mice control (*n* = 12, 13, 9, 6, 7 for each group, respectively). **d** The number of rearing exploration times within 5 min of old mice treated with vehicle or SSK1 compared with young, middle-aged, old mice control (*n* = 12, 13, 10, 9, 7 for each group, respectively). **e** Time to cross balance beam for old mice treated with vehicle or SSK1 compared with young, middle-aged, old mice control (*n* = 12, 13, 10, 9, 9 for each group, respectively). **f** Serum biochemical test. The levels of Alanine transaminase (ALT), aspartate transaminase (AST), creatinine (CREA), uric acid (UA) and carbamide (UREA) in old mice after vehicle or SSK1 treatment for 8 weeks are shown (Vehicle-treated, *n* = 8; SSK1-treated, *n* = 8). **g** Routine analysis of blood. The numbers of granulocytes (GR), lymphocytes (LY), white blood cells (WBC), red blood cells (RBC), and platelets (PLT) of old mice after vehicle or SSK1 treatment for 8 weeks are shown (Vehicle-treated, *n* = 8; SSK1-treated, *n* = 8). Each data point represents an individual mouse. ‘*n*’ represents the number of mice. Data are presented as means ± SEM. Unpaired two-tailed *t-*test, *P < 0.05, **P < 0.01.

Since SSK1 reduced chronic inflammation locally and systematically, we further tested the functional improvement in SSK1-treated aged mice. To this end, we evaluated their physical performance by a series of functional assays which were all declined in aged mice (Fig. 6a–e). Compared with vehicle treatment, SSK1 treatment for 8 weeks significantly increased the maximal rotarod time (Fig. 6a), treadmill distance (Fig. 6b), grip strength (Fig. 6c), and rearing exploration time (Fig. 6d) as well as decreased the balance beam crossing time (Fig. 6e) in aged female mice, without a significant effect on body weight (Supplementary information, Fig. S8a). Additionally, the treatment of SSK1 showed no obvious systemic toxicity from the results of the serum biochemical test and routine blood analysis (Fig. 6f, g). As a control, gemcitabine showed no obvious effect on physical function improvement (Supplementary information, Fig. S8g–j). The same results of SSK1 in rotarod, treadmill, and grip strength performance were also observed for aged male mice (Supplementary information, Fig. S8b–d). These results of mouse functional assays demonstrated an improvement in motor function, balance ability, exercise endurance, skeletal muscle capacity and spontaneous exploration. Notably, after SSK1 treatment, the performance of rotarod and beam balance was improved in aged mice compared with the pretreatment state (Supplementary information, Fig. S8e, f).

Additionally, we further compared SSK1 with other three reported senolytic drugs (ABT263, dasatinib plus quercetin, and fisetin) in their ability of improving physiological functions in aged mice.^11,12,15^ SSK1 improved the physical function in all four tested assays, but the effect of the other senolytic drugs was limited (Supplementary information, Fig. S8g–j). Fisetin increased the performance in two functional assays (grip strength and rearing frequency); ABT263 improved motor coordination and balance of mice in the rotarod test, while showed no obvious effect in other three assays; D + Q only led to a tendency of improvement in one functional assay (treadmill) (Supplementary information, Fig. S8g–j). Collectively, SSK1 could alleviate the dysfunction in all tested functional assays suggesting that SSK1 was more efficacious to improve physical function in aged mice.

To study the toxicological effects of SSK1 in vivo, we treated aged mice with SSK1 at a high concentration (100 mg/kg) and high frequency. Serum biochemical test and routine blood analysis showed no obvious systemic toxicities in these mice (Supplementary information, Fig. S9). As HUVECs showed compromised cell viability to some extend under SSK1 treatment in vitro (Supplementary information, Fig. S3i), we analyzed the in vivo toxicity of SSK1 to vessels in mice by performing CD31 immunostaining and TUNEL apoptosis staining. We hardly observed any apoptotic endothelial cells even upon treatment with high (3–100 mg/kg) dosages of SSK1 (data not shown).

We next analyzed the potential off-target effect of SSK1 in vivo, because elevated endogenous β-gal activity can also be found in some non-senescent cells.^48,49^ By analyzing *GLB1* expression in different tissues from different database,^50,51^ we found that the kidney and submandibular gland had higher expression of endogenous acidic β-gal, so we focused on analyzing the potential off-target effect of SSK1 in these two tissues. We found most β-gal-positive non-senescent cells were epithelium cells (Supplementary information, Fig. S10a, b), which contained Ki67-positive proliferating cells (Supplementary information, Fig. S10c). Notably, we found that only a few cells in the kidney were induced into apoptosis after SSK1 treatment (Supplementary information, Fig. S10d). Additionally, we found no obvious reduction of endogenous acidic β-gal-positive non-senescent cells after SSK1 treatment in the kidney and submandibular glands (Supplementary information, Fig. S11), further supporting the in vivo safety of SSK1 treatment. Collectively, our results suggested that SSK1-induced clearance of SA-β-gal-positive senescent cells to be a robust and safe approach to ameliorate age-related functional decline.

## Discussion

In this study, we designed a new prodrug based on the major senescence marker — the elevated β-gal — to selectively target senescent cells. This prodrug, SSK1, specifically killed both human and mouse senescent cells independent of senescent cell types and inducers. In a mouse lung injury model, SSK1 cleared stress-induced senescent cells in vivo and alleviated associated symptoms. Importantly, SSK1 also effectively reduced naturally occurring senescent cells in aged mice, decreased the senescence- and age-associated gene signatures, down-regulated SASP both locally and systemically, and restored physical functions. These results demonstrated the robustness and specificity of our prodrug in reducing senescent cells.

We successfully developed a new prodrug strategy that directed gemcitabine to kill senescent cells in a highly selective manner. The foundation of our prodrug strategy was to select a highly specific senescence marker and identify an appropriate potent drug to efficiently kill senescent cells. First, the elevated enzymatic activity of β-gal, a universal senescence biomarker both in vitro and in vivo,^18,52^ was utilized to direct the prodrug to specifically target senescent cells. Second, through the screening of hundreds of FDA-approved drugs, we found gemcitabine to be one of the most potent agents in killing non-dividing senescent cells (Fig. 1a; Supplementary information, Fig. S1b, c, and Table S1). In addition, gemcitabine is a widely used FDA-approved drug with proven safety and short plasma circulation time.^25,26^ Our designed prodrug SSK1 is activated to release gemcitabine selectively in senescent cells (Fig. 1c), following which senescent cells were effectively eliminated (Fig. 1d). Our further study showed that gemcitabine activates p38 to induce apoptosis in non-dividing senescent cell (Supplementary information, Fig. S2a, b). As a nucleoside analog, gemcitabine could kill senescent cells through inducing mitochondrial DNA damage (Supplementary information, Fig. S2d), similar to a reported nucleoside analog, Ganciclovir.^33^ These results demonstrated that prodrug SSK1 is superior to current reported senolytics in terms of design strategy and specificity.

Cellular senescence is a highly heterogeneous process due to the different cell origins and stimuli,^6,53^ whereas the key feature of SSK1 is the ability to efficiently clear senescent cells with a broad spectrum of cell types and senescence inducers, including replication, irradiation, oncogene and genotoxic stress (Fig. 2a, c). In this study, we compared SSK1 with other reported senolytics on HEFs, human preadipocytes and HUVECs, which were used to test senolytics. ABT263 (a classical anti-apoptosis BCL-2 inhibitor) eliminated senescent HEFs and HUVECs, but showed little effect on human preadipocytes (Supplementary information, Fig. S3b, f, j), which was also reported by other previous studies.^12,13^ The combination of dasatinib (a pan-tyrosine kinase inhibitor) and quercetin (a plant flavonoid) killed all three types of senescent cells in a dose-dependent manner (Supplementary information, Fig. S3c, g, k), but had a high toxic effect on non-senescent cells in consistence with others’ results.^11,16,36^ Another natural flavonoid fisetin, reported as a potential senotherapeutic agent,^15^ showed modest effect on eliminating senescent HEFs and preadipocytes even at higher concentrations (Supplementary information, Fig. S3d, h) which was comparable to other’s results.^54^ Most importantly, SSK1 could overcome these limitations, including cell-type dependency, high toxicity on non-senescent cells, and low efficiency on senescent cells (Supplementary information, Fig. S3a, e, i). Therefore, SSK1 possessed a better senolytic activity regarding of specificity and efficiency on a wider range of cell types, demonstrating the superiority of β-gal-based prodrug strategy to target senescence.

Moreover, we found SSK1 exerted its elimination effect on senescent cells in vivo. SSK1 eliminated stress-induced senescent cells effectively and decreased different senescence markers in bleomycin-induced lung injury model, highlighting its effectiveness in vivo (Fig. 3). SSK1 treatment also relieved lung fibrosis (Fig. 3d; Supplementary information, Fig. S4c, d) and attenuated the impaired physical function as tested by treadmill assay (Supplementary information, Fig. S4f). In this study, we also treated naturally aged mice with SSK1 and tested its effects on senescent cells, chronic inflammation and physical function. First, SSK1 could remove senescent cells in multiple tissues and decrease the senescence-associated signatures as shown by the GSEA analysis (Fig. 4b–g). Second, SSK1 could decrease the expression of SASP-associated genes in aged livers and kidneys and reduce chronic low-grade inflammation in the blood (Fig. 5a, b, e–h). Third, SSK1 ameliorated the impaired motor function, balance, exhausted exercise, muscle strength, and spontaneous exploration in aged mice (Fig. 6a–e). Most importantly, the performance of rotarod and beam balance in the SSK1-treated group was improved compared with that in the initial pretreatment condition (Supplementary information, Fig. S8e, f). Collectively, our prodrug SSK1 targeting β-gal-positive cells exerted very significant biological effects in naturally aged mice.

While SA-β-gal is widely used as a marker of cellular senescence,^20–22^ its elevated activity can be found in some other cells such as activated *macrophages*.^48,55^ These SA-β-gal-positive *macrophages* can be harmful and have been found to accumulate in injured and aged tissues contributing to chronic inflammation.^44,45^ Importantly, we have shown that SSK1 decreases the *number* of SA-β-gal-positive *macrophages* in injured lungs and aged livers (Supplementary information, Fig. S6g–j), which is consistent with our observation of reduced secretion of chronic inflammation-related cytokines. Therefore, eliminating macrophage accumulation by SSK1 might reduce chronic inflammation and benefit aged organisms. In addition, activated *macrophages* play crucial roles in acute inflammation and cytokine storm,^56^ especially those induced by virus infection. During virus-induced acute inflammation, *macrophages* produce pro-inflammatory factors and trigger initiation of cytokine storms.^57^ For instance, the depletion of *macrophages* could protect mice from coronavirus-induced lethal infection.^58^ Accordingly, activated *macrophages* could be potential targets for treating acute inflammation and cytokine storms via SSK1. The future potential for SSK1 in treatment of acute inflammation, injury, and age-induced chronic inflammation is promising.

An understanding of the toxicological effects of SSK1 in vivo is critical to clinical applications. Importantly, our data showed that high concentration (100 mg/kg) and high frequency SSK1 treatment had no apparent systemic toxicities (Supplementary information, Fig. S9). This provides strong evidence for the in vivo safety of SSK1. This safety profile is further supported by comparison to gemcitabine, an SSK1 effector and approved clinical drug. First, our effective in vivo dosage of SSK1 was approximately 60-fold lower than the clinical dosage of gemcitabine (30 mg/kg) and implies a greatly reduced risk of in vivo toxicity of SSK1.^24^ Second, gemcitabine is a nucleoside analog that potently affects rapidly dividing cells and the off-target effects of gemcitabine could be shown in different proliferating cell types.^59,60^ SSK1, however, targets only β-gal-positive cells. Notably, in addition to *macrophages*, we found that the majority of non-senescent β-gal-positive cells are rapidly dividing epithelial cells (Supplementary information, Fig. S10a, b). As a result, the potential types of proliferating cells with SSK1 sensitivity are greatly narrowed relative to gemcitabine. Even though small numbers of β-gal-positive epithelial cells are targeted by SSK1 treatment, these cells have robust self-renewal ability.^61^ Consistent with this self-renewal property, we found that epithelial tubular cells in the kidney with elevated β-gal activity had a high percentage of Ki67-positive cells (Supplementary information, Fig. S10c). Following SSK1 treatment, we observed only a few cells of this tubular cell population undergoing apoptosis (Supplementary information, Fig. S10d), and such low-level impairment could be easily repaired by the rapid self-renewal of tubular cells during SSK1 treatment. Moreover, the short duration of SSK1 treatment might avoid significant impairment in related tissues and further minimize its side effects. In summary, our study demonstrates the superiority and safety of this prodrug strategy by targeting β-gal to selectively remove senescent cells of multiple cell types. These findings open a new avenue for the treatment of age-associated diseases and provide a clinical opportunity for intervention into the aging process.

## Materials and methods

### Synthesis of SSK1

Unless otherwise mentioned, all reactions were carried out under a nitrogen atmosphere with dry solvents under anhydrous conditions. Reagents were purchased at the highest commercial quality and used without further purification, unless otherwise stated. Yields were measured chromatographically.

Reactions were monitored by thin-layer chromatography on plates (GF254) supplied by Yantai Chemicals (China) using UV light as the visualizing agent and an ethanolic solution of phosphomolybdic acid and cerium sulfate and heat as the developing agents. Unless otherwise specified, flash column chromatography used silica gel (200–300 mesh) supplied by Tsingtao Haiyang Chemicals (China).

NMR spectra were recorded on a Brüker Advance 600 (^13^C 150 MHz, ^19^F 565 MHz) and a Brüker Advance 400 (^1^H 400 MHz) spectrometers, which were calibrated using residual undeuterated solvent (CD_3_OD at 3.31 ppm ^1^H NMR, 49.0 ppm ^13^C NMR). The following abbreviations were used to explain the multiplicities: s = singlet, d = doublet, t = triplet, dd = doublet of doublets, m = multiplet.

Mass spectrometric data were obtained using a Brüker Apex IV FTMS using ESI (electrospray ionization).



Gemcitabine (2.0 equiv, 0.340 mmol, 89.5 mg) and DIPEA (2.4 equiv, 0.402 mmol, 70 μL) were dissolved in 4 mL of DMF. The mixture was stirred for 20 min in a microwave reaction vessel before addition of 1 (1 equiv, 0.170 mmol, 113 mg).^62^ The reaction was placed in a CEM Discover microwave reactor (100 °C) and irradiated for 45 min. Then, DMF was removed in vacuo, and the residue was purified by flash column chromatography to give the desired product SSK1 (39.5 mg, 29% yield) as a white solid.

*R*_*f*_ = 0.3 (dichloromethane: acetone = 2:1); ^1^H NMR (400 MHz, CD_3_OD): δ 8.31 (d, *J* = 7.6 Hz, 1 H), 7.90 (s, 1 H), 7.69 (d, *J* = 8.6 Hz, 1 H), 7.47 (d, *J* = 8.6 Hz, 1 H), 7.31 (d, *J* = 7.6 Hz, 1 H), 6.24 (t, *J* = 7.2 Hz, 1 H), 5.47 (d, *J* = 3.4 Hz, 1 H), 5.39 (d, *J* = 3.3 Hz, 1 H), 5.38 (s, 1 H), 5.27–5.24 (m, 3 H), 4.35 (t, *J* = 6.5 Hz, 1 H), 4.32–4.26 (m, 1 H), 4.21 (d, *J* = 6.4 Hz, 2 H), 3.99–3.94 (m, 2 H), 3.81 (dd, *J* = 12.9, 3.2 Hz, 1 H), 2.18 (s, 3 H), 2.08 (s, 3 H), 2.04 (s, 3 H), 1.97 (s, 3 H); ^13^C NMR (150 MHz, CD_3_OD) δ 172.01, 171.93, 171.41, 171.26, 165.22, 157.29, 154.18, 150.26, 145.72, 142.22, 134.66, 132.87, 125.72, 123.89 (t, *J* = 258.9 Hz), 119.81, 101.03, 97.02, 86.34 (t, *J* = 32.0 Hz), 82.76 (t, *J* = 3.8 Hz), 72.58, 71.96, 70.14 (t, *J* = 23.0 Hz), 69.50, 68.56, 66.90, 62.60, 60.25, 20.69, 20.65, 20.50, 20.49; ^19^F NMR (565 MHz, CD_3_OD) δ -119.06 (dd, *J* = 239.7, 9.8 Hz, 1 F), −119.91 (d, *J* = 241.7 Hz, 1 F); HRMS-ESI (m/z): calc’d for C_31_H_35_F_2_N_4_O_18_ [M + H^+^] 789.1909, found 789.1907.

### Cell culture

The mouse primary cells (MEFs, NBFs, and adult mouse lung fibroblasts) were cultured in high-glucose DMEM (Gibco) supplemented with 10% fetal bovine serum (FBS, VISTECH) and 1% penicillin-streptomycin (Gibco). HEFs were cultured in medium Dulbecco’s modified Eagle’s medium (DMEM, Gibco) containing 10% fetal bovine serum (FBS, VISTECH), 1% GlutaMAX (Gibco), 1% Non-Essential Amino Acids (NEAA, Gibco) and 1% penicillin/streptomycin (Gibco). HUVECs were cultured in Endothelial Cell Medium (ECM, sciencecell) with 10% FBS and 1% penicillin/streptomycin (Gibco). Human preadipocytes were cultured in Mesenchymal Stem Cell Growth Medium 2 (PromoCell, C-28009). All the cells were all cultured in a humidified incubator at 37 °C and 5% CO_2_.

Mouse embryonic fibroblasts (MEFs) were isolated from E13.5 embryos as described previously.^63^ Newborn mouse skin fibroblasts (NBFs) were isolated from the skin of day 1–3 newborn mice. Adult mouse lung fibroblasts were isolated from 3-month-old mice. Briefly, mouse embryonic tissues, skin and lung tissues were obtained from described donor mice. Then, these tissues were minced with forceps and incubated in 2 mg/mL collagenase IV (Gibco) for 2–4 h at 37 °C. After enzyme treatment, cells were collected by centrifugation and resuspended in high-glucose DMEM (Gibco) supplemented with 10% (VISTECH) and 1% penicillin-streptomycin (Gibco).

For human primary fibroblast isolation, the present study was approved by the Research Ethics Committee of China-Japan Friendship Hospital (Ethical approval No: 2009-50) and the Institute of Review Board in Peking University (IRB 00001052-1508719070) and conducted according to the approved protocol. Samples were collected from consenting donors according to ethically approved procedures at China–Japanese Friendship Hospital. Human embryonic skin fibroblasts (HEFs) were obtained as previously described.^64^ Briefly, human embryonic skin tissues, obtained from aborted tissues with informed patient consent, were minced with forceps and incubated in 1 mg/mL collagenase IV for 1–2 h at 37 °C. After enzyme treatment, cells were collected by centrifugation and resuspended in HEF medium (DMEM containing 10% FBS, 1% GlutaMAX, 1% NEAA and 1% penicillin/streptomycin). Cells were plated on 10 cm tissue culture dishes and grown in HEF medium.

HUVECs were purchased from Lonza (C2519A) and cultured in Endothelial Cell Medium (sciencecell, 1001). HUVECs were subcultured and cryopreserved according to the protocol from Lonza.

Human preadipocytes, were obtained as previously described.^65^ Briefly, human adipose tissues were collected from patients during liver or gallbladder surgery. The samples were washed with phosphate-buffered saline (PBS) containing 5% Penicillin/Streptomycin (P/S) to remove visible blood vessels and debris. Then, the tissues were minced with forceps and digested with 1 mg/mL Collagenase Type IV containing 2% P/S for 2–4 h at 37 °C, 5% CO_2_. After enzyme treatment, cells were collected by centrifugation and resuspended in DMEM (Gibco) containing 10% fetal bovine serum (FBS, VISTECH) and 1% penicillin/streptomycin (Gibco). Cells were plated on 10 cm tissue culture dishes and grown in Mesenchymal Stem Cell Growth Medium 2 (PromoCell, C-28009).

### Cellular senescence induction

To avoid replication-induced senescent cells, low-passage proliferative NBFs (<3 passages), MEFs (<3 passages), mouse lung fibroblasts (<3 passages), HEFs (<10 passages), HUVECs (<6 passages), and human preadipocytes (<5 passages) were used as normal controls.

For replication-induced senescence, cells were passaged until they lost the ability to proliferate and became senescent. NBFs and MEFs were used as senescent cells after approximately 8–9 passages or 12 population doublings. Mouse lung cells were senescent after approximately 7 passages or 10 population doublings. HEFs were used after approximately 35 passages or 50 population doublings. HUVECs were senescent after approximately 18 passages or 24 population doublings. Human preadipocytes were senescent after approximately 10 passages or 15 population doublings.

For ionizing radiation-induced senescence, MEFs and HEFs were exposed to 10 Gy of ionizing irradiation in an RS 2000 X-ray Biological Irradiator (Rad Source Technologies, Inc.) at a dose rate of 1.205 Gy/min. The day after irradiation, MEFs, HEFs and HUVECs were passaged at a 1:2 dilution and cultured for another three days. Then these cells were plated to carry out further experiments.

For oncogene-induced senescence by ectopic expression of KRAS, cells were transfected with lentivirus carrying pLenti-PGK-KRAS4B (G12V) or control vector (Addgene, 35633). One day after viral infection, the medium was exchanged, and transfected cells were cultured for another 3 days and passaged at a 1:3 dilution. After another 3 days of culture, the cells became senescent enough for further analysis.

For etoposide-induced senescence, MEFs were treated with 2 µM etoposide for 12–18 h, and HEFs were treated with 5 µM etoposide for 12–18 h. Three days after treatment, cells were sub-cultured at a 1:3 ratio for 3 days in a fresh medium before further analysis.

For peroxide hydrogen (H_2_O_2_)-induced senescence, cells were incubated in medium with 200 μM H_2_O_2_ for 4 h or 100 μM for 12 h and cultured in fresh medium for several days until they lost the capacity of proliferation.

### Cell viability testing

To test the viability of senescent and non-senescent cells treated with SSK1, cells were stained with a mixture of Hoechst 33342 and propidium iodide (PI) (Solarbio, CA1120) to distinguish living and dead cells. After 3 days of treatment with the small molecules, the cell culture medium was removed, and the cells were washed once with PBS. Then, the cells were stained according to the manufacturer’s protocol, where the final concentrations of Hoechst 33342 and PI were 5 μg/mL and 2 μg/mL in PBS buffer. The plate was incubated at 4 °C for 30 min and observed on a fluorescence microscope or automatic cell imaging system. Viable cells were quantified using a MD Image Xpress Micro XL (Molecular Devices). The treatments include the following small molecules: gemcitabine (MedChem Express, HY-17026), SSK1 alone, SSK1 with SB203580 (MedChem Express, HY-10256), SSK1 with SB202190 (MedChem Express, HY-10295), SSK1 with Birb796 (MedChem Express, HY-10320), SSK1 with Dipyridamole (Yuanye, B25482), LY2334737 (Targetmol, T4061), 4-(Hydroxymethyl)-2-nitrophenol (Bidepharm, BD97717), ABT-263 (Selleck, S1001), Dasatinib (Selleck, S1021) plus Quercetin (Selleck, S2391), Fisetin (Selleck, S2298).

### FDA-approved drug screening

Replication-induced senescent Newborn mouse skin fibroblasts (NBFs) were seeded in 24-well plates and treated with an FDA-approved drug screening library (selleckchem). Compounds in the library were diluted to a final concentration of 1 μM. After 3 days of treatment, the cell viability was tested.

### SSK1 metabolism analysis

To detect the release of gemcitabine from SSK1 in senescent and non-senescent cells, the metabolism analysis was performed as previously reported.^66^ Briefly, the senescent and non-senescent cells were incubated with 0.5 μM SSK1 in DMEM medium containing 10% FBS and 1% penicillin-streptomycin for the indicated time. Then the cells were slightly washed with PBS for three times, then harvested and counted. Cold methanol used to extract the compounds and samples were prepared after centrifugation. The concentration of gemcitabine was determined by LC-MS/MS analysis.

### Flow cytometry analysis

To test apoptosis of cells treated with Vehicle or SSK1 by flow cytometry analysis, NBFs were digested into single-cell suspension by incubation with 0.25% trypsin-EDTA (Invitrogen) at 37 °C for 3–5 min or with accutase at 37 °C for 10 min. Cells were then stained with FITC annexin V and propidium iodide (PI) according to the manufacturer’s protocol of Annexin V-FITC Apoptosis Detection Kit (Beyotime Biotechnology, C1063). Flow cytometry was performed within 1 h on an Aria III (BD Biosciences) or FACSVerse (BD Biosciences). Data were analyzed using FlowJo software (FlowJo LLC). Viability was calculated as the percentage of PI and Annexin V double-negative cells. Apoptotic cells were Annexin V-positive cells.

To test the SA-β-gal activity by flow cytometry analysis, senescent and non-senescent cells were digested into single cells by trypsinization, and then incubated with 33 μM 5-dodecanoylaminofluorescein di-β-D-galactopyranoside (C12FDG) (Invitrogen, I-2904) for 20–60 min. Pre-treatment with chloroquine diphosphate may inhibit endogenous β-galactosidase activity. The cells were washed with PBS after staining and the cell suspensions were run immediately in a FACSVerse (BD Biosciences). Data were analyzed using FlowJo software (FlowJo LLC).

To test β-gal-positive cell types in bleomycin-induced lung injury model and mice kidneys and salivary glands by flow cytometry analysis, we first treated the isolated primary cells with 33 μM C12FDG-containing culture medium for 20–60 min. Then these cells were co-stained by cell type markers on ice for 45 min. The monoclonal conjugated antibodies were used: anti-CD45 PerCP-Cy5.5 (1:200, eBioscience, 45-0451-82), anti-CD326 (EpCAM) PE-Cy7 (1:200, eBioscienc, 25-5791-80). DAPI (Beyotime, C1002) was used for live/dead discrimination. All samples were acquired and sorted using an FACSVerse (BD Biosciences). Data were analyzed using FlowJo software (FlowJo LLC).

### Cell proliferation assays

For BrdU staining, cells were labeled with BrdU (5 μM in culture medium) (Sigma-Aldrich, B5002) for 3–12 h and then fixed with 4% paraformaldehyde (DingGuo, AR-0211). Cells were incubated in 2 M HCl for 1 h and neutralized with 0.1 M sodium borate buffer pH 8.5 for 15 min. BrdU incorporation was measured by immunofluorescence staining (IF).

For EdU staining, cells were labeled with EdU (10 μM) for 3 h and stained according to the manufacturer’s protocol (Beyotime, C0071S). BrdU or EdU incorporation was quantified by high content analysis microscopy — MD Image Xpress Micro XL (Molecular Devices).

### Reverse transcription (RT)-quantitative PCR (RT-qPCR)

Total RNA was isolated using the Direct-zol RNA MiniPrep Kit (Zymo Research, R2072). RNA was treated with DNase and converted to cDNA using TransScript First-Strand cDNA Synthesis SuperMix (TransGen Biotech, AT311–03). RT-qPCR was performed using Kapa SYBR® FAST qPCR Kit Master Mix (Kapa Biosystems, KM4101) on a CFX Connect^TM^ Real-Time System or CFX96^TM^ Real-Time System (Bio-Rad). Data were analyzed using the 2^(−ΔΔCt)^ method. *GAPDH* was used as a control to normalize the expression of target genes.

### Primers for specific genes of mouse

**Table:** 

Gene	Forward Primer (5′ to 3′)	Reverse Primer (5′ to 3′)
*p16*	GCCCAACGCCCCGAACTCTTTC	GCGACGTTCCCAGCGGTACACA
*p21*	CCTGGTGATGTCCGACCTG	CCATGAGCGCATCGCAATC
*p19*	GCCGCACCGGAATCCT	TTGAGCAGAAGAGCTGCTACGT
*IL1α*	AAGTCTCCAGGGCAGAGAGG	CTGATTCAGAGAGAGATGGTCAA
*IL1β*	AAAAGCCTCGTGCTGTCG	AGGCCACAGGTATTTTGTCG
*IL6*	GTTCTCTGGGAAATCGTGGA	GGTACTCCAGAAGACCAGAGGA
*CXCL1*	ACCGAAGTCATAGCCACACTC	CTCCGTTACTTGGGGACACC
*TNFα*	GCCTCTTCTCATTCCTGCTT	CTCCTCCACTTGGTGGTTTG
*GLB1*	GGATGGACAGCCATTCCGAT	CAGGGCACGTACATCTGGATA
*Fn1*	CCACCCCCATAAGGCATAGG	GTAGGGGTCAAAGCACGAGTCATC
*Col1a1*	TGCCGTGACCTCAAGATGTG	CACAAGCGTGCTGTAGGTGA
*Col3a1*	GCGGAATTCCTGGACCAAAAGGTGATGCTG	GCGGGATCCGAGGACCACGTTCCCCATTATG
*GAPDH*	CTTTGTCAAGCTCATTTCCTGG	TCTTGCTCAGTGTCCTTGC

### Primers for specific genes of human

**Table:** 

Gene	Forward Primer (5′ to 3′)	Reverse Primer (5′ to 3′)
*p16*	ATATGCCTTCCCCCACTACC	CCCCTGAGCTTCCCTAGTTC
*GAPDH*	TGACATCAAGAAGGTGGTGAAGCAGG	GCGTCAAAGGTGGAGGAGTGGGT

### Plasmid construction and lentivirus production

Plasmids used to interfere the expression of *GLB1* with short hairpin RNAs (shRNAs) were obtained from Sigma MISSION shRNA. Genetic knockdown was performed according to the manufacturer’s protocol. The shRNA sequences are listed below. Lentivirus production, collection, and infection were as described.^67^

### shRNA sequences

**Table:** 

shRNA	Sequences (5′ to 3′)
sh*GLB1*#1	CCGGGAGAAGTCATTCAGATGTTTACTCGAGTAAACATCTGAATGACTTCTCTTTTTTG
sh*GLB1*#2	CCGGGTGCTAGAATGGAAGCTACATCTCGAGATGTAGCTTCCATTCTAGCACTTTTTTG
sh*GLB1*#3	CCGGGAGAAGTCATTCAGATGTTTACTCGAGTAAACATCTGAATGACTTCTCTTTTTTG
shControl	CCGGCAACAAGATGAAGAGCACCAACTCGAGTTGGTGCTCTTCATCTTGTTGTTTTTG

### Western blot

Senescent and non-senescent NBFs were plated and incubated overnight at 37 °C. The cells were treated with 0.05 μM gemcitabine or 0.5 μM SSK1 for the indicated time. Before harvest, cells were washed twice with pre-cooled PBS buffer. Total protein was extracted with lysis buffer (50 mM Tris-HCl (pH 7.5), 137 mM sodium chloride, 1 mM EDTA, 1% Nonidet P-40, 10% glycerol, 0.1 mM sodium orthovanadate, 10 mM sodium pyrophosphate, 20 mM β-glycerophosphate, 50 mM sodium fluoride, 1 mM phenylmethylsulfonyl fluoride), and the protein concentrations were normalized using the BCA Protein Assay Kit (Thermo Fisher Scientific, 23225). Protein samples were mixed with protein loading buffer and incubated at 95 °C for 5 min. Western blot was performed by using the following antibodies: phospho-specific p38 MAPK (Thr180/Tyr182) (Cell Signaling Technology, 4511) and p38 (Cell Signaling Technology, 8690); phospho-MKK3(S189)/MKK6(S207) (Cell Signaling Technology, 9231); cleaved caspase 3 (Cell Signaling Technology, 9661). The β-tubulin or β-actin protein level was also determined as the loading control by using the β-tubulin antibody (Cell Signaling Technology, 2128) or β-actin antibody (Cell Signaling Technology, 4970).

### Mitochondrial DNA analysis

About 0.5 × 10^7^ senescent or non-senescent cells were treated with 0.5 μM SSK1 or DMSO in this assay. After treatment, the cells were washed with PBS and collected for mitochondrial DNA extraction by using the kit (Abcam, ab65321). The concentration of mitochondrial DNA was measured by nanodrop. Then 500 ng mitochondrial DNA from each sample was used and separated by 1% agarose gel for analysis.

### Mice

All animal experiments were performed according to the Animal Protection Guidelines of Peking University, China. C57BL/6 (C57) mice were obtained from Beijing Vital River Laboratory Animal Technology Co, Ltd. and maintained under specific pathogen-free facility (SPF) conditions with a 12 light/12 dark cycle and free access to food and water. Young mice were 2–4-month-old; middle-aged mice were 9–12-month-old; old mice were 20–24-month-old. Old male mice were caged individually, and female mice and young male littermates were maintained with no more than five mice per cage.

### Bleomycin-induced lung injury

Bleomycin was purchased from Selleck (S1214). To induce lung senescence and fibrosis, male and female mice (3–6-month-old) were subjected to transtracheal injection of bleomycin (1.5 mg/kg) as previously reported.^68^

### Drug treatments

All drugs were mixed in 90% PBS, 5% Tween-80 (Sigma, P1754), and 5% polyethylene glycol (PEG) (Sigma, 81172) and administered to mice by intraperitoneal (i.p.) injection. For bleomycin-treated young mice, SSK1 (0.5 mg/kg) or vehicle were given consecutive two days every week for four weeks. For old mice (20-month old), SSK1 (0.5 mg/kg), gemcitabine (0.5 mg/kg), ABT263 (5 mg/kg), dasatinib (1 mg/kg) plus quercetin (10 mg/kg), fisetin (20 mg/kg) or vehicle (DMSO) were administrated for continued 3 days every 2 weeks for 8 weeks. Mice were randomized to SSK1, gemcitabine or vehicle delivered by intraperitoneal injection.

### Toxicity testing in vivo

Old mice were injected with vehicle and SSK1 (3, 10, 30, 60, 100 mg/kg) 3 times a week for 5 weeks. After treatment, the blood samples were collected for further analysis.

### SA-β-galactosidase staining of cultured cells and frozen sections

Cultured cells were washed once with PBS and fixed in SA-β-galactosidase (SA-β-gal) staining fix solution for 15 min at room temperature. Cells were then washed three times with PBS and incubated with SA-β-gal staining solution (Beyotime Biotechnology, C0602) for 16–20 h at 37 °C. For 12-well plates, 1 mL of β-gal staining solution was added to each well. The plate was sealed with Parafilm to prevent evaporation of the staining medium. After the overnight incubation, cells were washed with PBS and observed under a bright field microscope.

For SA-β-gal staining, the frozen sections were dried at 37 °C for 20–30 min and then fixed in SA-β-gal staining fix solution for 15 min at room temperature. The frozen sections were washed three times with PBS and incubated with SA-β-gal staining solution (Beyotime Biotechnology, C0602) overnight at 37 °C. After completion of SA-β-gal staining, the sections were stained with eosin for 1–2 min, rinsed under running water for 1 min, differentiated in 1% acid alcohol for 10–20 s, and washed again under running water for 1 min. Sections were dehydrated in increasing concentrations of alcohols and cleared in xylene. Excess xylene was drained and a coverslip was placed over the section. After drying, the sample was observed under a microscope.

Lung, liver, and kidney frozen sections stained with SA-β-gal were quantified by ImageJ software (NIH) to measure the SA-β-gal-positive area. The total area was quantified by eosin-positive area. The relative SA-β-gal-positive cells were calculated with the SA-β-gal-positive area divided by the total area. For the statistics of SA-β-gal-positive area of lung, the regions of lung NBFs were randomly selected to be photographed, avoiding analysis of larger pulmonary blood vessels and trachea. For the statistics of SA-β-gal-positive area of liver, the regions were randomly selected to be photographed. For the statistics of SA-β-gal-positive area of kidney, the regions of renal cortex were randomly selected to be photographed. Each tissue was measured with 10–18 regions.

### β-galactosidase of frozen sections

For endogenous acidic β-galactosidase (β-gal) staining, the kidney and salivary gland frozen sections were dried at 37 °C for 20–30 min and then fixed in β-gal staining fix solution for 15 min at room temperature. The frozen sections were washed three times with PBS and incubated with β-gal staining solution (Beyotime Biotechnology, C0605) overnight at 37 °C. The following protocol was similar to that of the SA-β-gal staining.

### Masson’s trichrome stain

To test the fibrosis in lung or liver tissues, the Masson’s trichrome staining kit (Solarbio, G1340) was used. The formalin-fixed, paraffin-embedded sections of lungs or livers were defaraffinized with xylene and rehydrated through 100% alcohol, 95% alcohol and running tap water. Then the sections were stained according to the manufacturer’s instruction. The sections were dehydrated very quickly through 95% alcohol and 100% alcohol, cleared by xylene and then covered with cover slides. The images were collected using a bright-light microscope (10X magnification objective) (OLYMPUS, BX43) and analyzed by ImageJ software (NIH) to measure the fibrosis area and total area. Each tissue was measured with 5 different regions. The percentage of fibrosis was calculated with the fibrosis-positive area divided by the total area.

### Immunofluorescence staining

Mouse tissues were fixed in 4% paraformaldehyde (DingGuo, AR-0211) for 24 h at room temperature and dehydrated with graded sucrose solution (20% and 30% respectively 24 h) before embedded in OCT compound (Sakura, 4583) for cryosection. The embedded tissues were cut and affixed on slides. Before immunohistochemical staining, the sections were fixed in 4% paraformaldehyde (DingGuo, AR-0211) at room temperature for 15 min and blocked with PBS containing 0.3% Triton X-100 (Sigma-Aldrich, T8787) and 2% normal donkey serum (Jackson ImmunoResearch Laboratories, 017-000-121) at room temperature for 1 h. Samples for immunofluorescence staining were incubated with primary antibody at 4 °C overnight, washed three times with PBS and then incubated with appropriate secondary antibodies for 1.5 h at 37 °C. Nuclei were stained with Hoechst 33342 (Sigma-Aldrich, B2261).

To identify the cell type of SA-beta-gal-positive cells, we co-stained SA-beta-gal with different cell type markers by immunofluorescence staining, the primary antibodies used in this study including: anti-F4/80 antibody (Abcam, ab6640) for detecting macrophage, anti-S100A4 (Fibroblast-specific protein 1, FSP1) antibody (Abcam, ab197896) for detecting fibroblasts, anti-EpCAM antibody (Abcam, ab71916) for detecting epithelial cells, anti-CD31 antibody (Abcam, ab28364) for detecting endothelial cells. To identify senescent cells, the primary antibody p21 (Abcam, ab188224) was used. To identify proliferative cells, the primary antibody Ki67 (MilliporeSigma, AB9260) was used.

The secondary antibodies were Alexa Fluor® 488 AffiniPure Donkey Anti-Rabbit IgG (H + L) (Jackson ImmunoResearch Laboratories, 71-545-152), Alexa Fluor® 488 AffiniPure Donkey Anti-Rat IgG (H + L) (Jackson ImmunoResearch Laboratories, 712-545-150), and Cy™3 AffiniPure Donkey Anti-Rabbit IgG (H + L) (Jackson ImmunoResearch Laboratories, 711-165-152). For quantification of p21-positive cells, images were randomly taken at 20× magnification at the same exposure using confocal laser endomicroscopy (LSM 710 NLO &DuoScan System) and then analyzed by ImageJ. Each mouse was measured with at least 10 regions.

### RNA sequencing

Total RNA was isolated using Direct-zol RNA MiniPrep Kit (Zymo Research). RNA sequencing libraries were constructed using the NEBNext Ultra RNA Library Prep Kit for Illumina (NEB England BioLabs). Fragmented and randomly primed 2 × 150 bp paired-end libraries were sequenced using Illumina HiSeq X Ten. RNA sequencing and raw data quality control were performed by Novogene Co., Ltd.

The sequencing data quality were checked by FastQC (version 0.11.8, http://www.bioinformatics.babraham.ac.uk/projects/fastqc/). Sequencing data were aligned to the mm10 reference genome by TopHat2. For differential gene expression analysis, read count of each gene was obtained by HTSeq^69^ (version 0.11.1, https://htseq.readthedocs.io/en/release_0.11.1/). The leading log-fold-change of different conditions which was calculated by R package edgeR^70^ (version 0.38.0, https://bioconductor.org/packages/release/bioc/html/edgeR.html) was used as input for gene set enrichment analysis. The gene sets of mouse molecular signature were obtained by msigdbr (version 7.0.1, https://cran.r-project.org/web/packages/msigdbr/index.html). In this package, the original human genes of Molecular Signatures Database (MSigDB v7.0, http://software.broadinstitute.org/gsea/msigdb/index.jsp) were converted to non-human model organism homologous genes. Gene set enrichment analysis was performed by clusterProfiler^71^ (version 3.14.0) (https://bioconductor.org/packages/release/bioc/html/clusterProfiler.html). R (version 3.6.0, https://cran.r-project.org/) were used for gene expression analysis.

Gene ontology (GO) term enrichment analyses for Supplementary information, Fig. S7a-f were performed using DAVID 6.8 functional annotation tool.^72^ The gene lists were selected by comparing gene expression between old and young mice or SSK1-treated and vehicle-treated old mice with *t-*test statistics: fold changes > 2 and *P* values < 0.05. The top five Go terms of GO_Biological Progress and GO_Cellular Component were shown in Supplementary information, Fig. S7a–f.

### ELISA analysis

Mouse blood samples were collected, stewed 2 h at room temperature or overnight at 4 °C, and then centrifuged (3000 rpm, 10 min) to gain serum. Secretion of mouse IL1α, IL6, CXCL1 and TNFα was measured using Mouse Interleukin 1α (IL1α) ELISA Kit (CUSABIO, CSB-E04621m), Mouse Tumor Necrosis Factor α (TNFα) ELISA Kit (CUSABIO, CSB-E04741m) kit, Mouse chemokine (C-X-C motif) ligand 1 (CXCL1) ELISA kit (CUSABIO, CSB-E17286m) and IL-6 (Interleukin-6) Mouse ELISA Kit (Abcam, ab100712) according to the manufacturers’ instructions.

### Physical function measurements

All functional assays were conducted at least 5 days after the last dose of drug was administered.

The rotarod test was used to evaluate motor coordination and balance with an accelerating RotaRod system (SANS Bio Instrument, SA102). Mice were placed in separate lanes on the rod rotating at an initial speed of 4 rpm/min. The apparatus was set to accelerate from 4 to 44 rpm/min in 300 s. A timer was used to record when each mouse fell or clung to the rod and completed a full passive rotation. Mice were trained at least two times on days 1 and 2 and tested on days 3, 4, and 5. Results were the average over 3 trials.

Treadmill exhaustion tests were used to evaluate exercise capacity and endurance. A motorized treadmill was used at an incline of 5° with 0.5 mA electrical stimulation (SANS Bio Instrument, SA102). Mice were trained for three days, starting at an initial speed at 5 m/min for 2 min and accelerating to 7 m/min for 2 min and then 9 m/min for 1 min. After three training sessions and one day of rest, mice were tested on the fifth day at an initial speed of 5 m/min, which increased by 2 m/min every 2 min until mice were unable to return to the treadmill. The distance (m) traveled before exhaustion was recorded for each mouse.

Mice were placed on the top of a grid strength meter (Columbus Instruments, 1027DM), so they grasped the grid with all four paws. The meter was set to the Peak Tension (T-PK) mode and recorded the grip strength over seven trials. The grip strength (N) was averaged, with the maximum and minimum data points excluded.

Mice were placed on a 1-m long, 6-mm wide beam resting 60 cm above the floor. A black box full of nesting material from the home cage was placed at the end of the beam apparatus as the end point. At the first day, mice were trained three times to walk across the beam to the safe box successfully without hesitation or observation. On the test day, the time (s) to cross the center 80 cm mark was measured by two motion detectors: one at 0 cm that starts a timer and one at 80 cm that stops the timer.

Mice were taken from the housing room into the testing room and allowed to acclimate to the new environment for a minimum of 30 min before the test. Mice were carefully placed in a 14-cm high and 11-cm diameter transparent cylinder and recorded for 5 min by video camera. The resulting video was analyzed to measure the rearing frequency (when mice stand only on hind legs, raise forelimbs off the ground, and stand upright for over 1 s).

### Blood analysis

For blood routine examination, 50 μL fresh blood was collected from each mouse and mixed with EDTA immediately. The blood samples were analyzed by Celltac Alpha MEK-6400 series hematology analyzers (Nihon Kohden, MEK-6400). For serum biochemical analysis, blood samples were collected, clotted for 2 h at room temperature or overnight at 4 °C, and then centrifuged (1000× *g*, 10 min) to obtain serum. 200 μL serum was aliquot and analyzed of Alanine transaminase (ALT), aspartate transaminase (AST), uric acid (UA) and creatinine (CREA) by Chemistry Analyzer (Mindray, BS-350E).

### TUNEL Apoptosis analysis

To test the off-target effect of SSK1 on tissues, we examined the apoptosis of cells in kidneys. The experiment was performed according to the manufacturer’s protocol (Beyotime, C1090).

### Statistical analyses

For statistical analyses, *P* values were calculated by *t-*test (when comparing only two groups) or one-way ANOVA or two-way ANOVA (when comparing more than two groups) in Excel or GraphPad Prism 8 with default parameters. All results are expressed as the mean ± SEM, and *n* indicates the number of experiment replicates or the number of mice. P values are as follows: *P < 0.05; **P < 0.01; ***P < 0.001; ****P < 0.0001.

## Supplementary information

Supplementary information Figure S1

Supplementary information Figure S2

Supplementary information Figure S3

Supplementary information Figure S4

Supplementary information Figure S5

Supplementary information Figure S6

Supplementary information Figure S7

Supplementary information Figure S8

Supplementary information Figure S9

Supplementary information Figure S10

Supplementary information Figure S11

Supplementary information, Table S1

Supplementary information, Table S2

Supplementary information, Table S3
