# Supplementary material for: Elimination of senescent cells by β-galactosidase-targeted prodrug attenuates inflammation and restores physical function in aged mice
Source: Cell Res. 2020 Apr 27;30(7):574–89. doi: 10.1038/s41422-020-0314-9 (PMC7184167; doi:10.1038/s41422-020-0314-9)
Supplement: Supplementary file 1 — Supplementary information Figure S1 [file 41422_2020_314_MOESM1_ESM.pdf]

Supplementary information, Figure S1

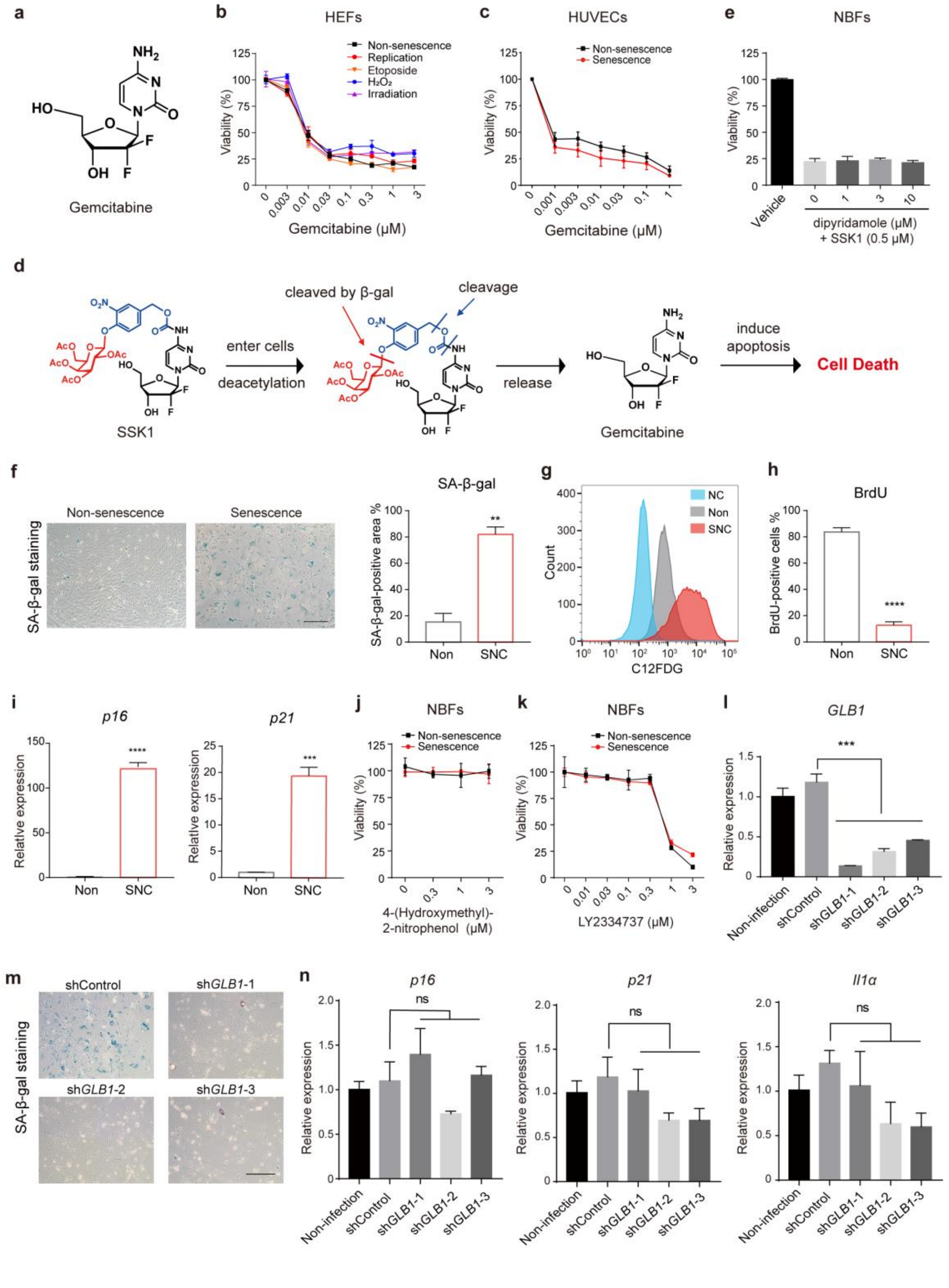

Supplementary information Fig. 1: Design of SSK1 and verification of *GLB1* knockdown.

a Molecular structure of gemcitabine. **b** Quantification of cell viability of different stimuli-induced senescent HEFs incubated with increasing concentrations of

gemcitabine for 3 days (*n* = 3). **c** Quantification of cell viability of non-senescent and replication-induced senescent HUVECs incubated with increasing concentrations of gemcitabine for 3 days (*n* = 4). **d** Model of SSK1 selectively killing senescent cells. **e** Quantification of cell viability of senescent NBFs incubated with vehicle (DMSO) or increasing concentrations of transporter inhibitor dipyridamole with SSK1 (0.5 μM) for 3 days (*n* = 3). **f** Representative images (**left**) and quantification (**right**) of SA-β-gal staining of non-senescent and replication-induced senescent NBFs (*n* = 3). Scale bar, 200 μm. **g** Representative flow cytometric histogram detecting SA-β-gal activity using C12FDG in non-senescent (Non) and senescent (SNC) NBFs. NC: unstained negative control. **h** Percentage of BrdU-positive cells in non-senescent and replication-induced senescent NBFs (*n* = 3). **i** RT-qPCR to measure expression of *p16* (**left**) and *p21* (**right**) in non-senescent and replication-induced senescent NBFs (*n* = 3). **j** Quantification of viability of non-senescent and replication-induced senescent NBFs incubated with the indicated concentrations of 4-(hydroxymethyl)-2-nitrophenol for 3 days (*n* = 3). **k** Quantification of viable cells in non-senescent and replication-induced senescent NBFs incubated with the indicated concentrations of gemcitabine-based prodrug LY2334737 for 3 days (*n* = 3). **l** RT-qPCR to measure gene expression of *GLB1* in the shControl and sh*GLB1* knockdown treatments (*n* = 3). **m** SA-β-gal staining of shControl and sh*GLB1*-1, -2 and -3 knockdown treatments. Scale bar, 200 μm. **n** RT-qPCR to measure expression of *p16, p21* and *Il1α* in the shControl and sh*GLB1* knockdown treatments (*n* = 3). For cell viability analysis in **(b), (c), (e), (j)** and **(k)**, cell numbers were quantified using Hoechst 33342 staining and dead cells were excluded by PI staining, and then cell viability was plotted. Data are presented as means ± SEM. '*n*' represents number of biological replicates. Unpaired two-tailed *t*-test for (**f**), (**h**) and (**i**), two-way ANOVA test for (**l**) and (**n**), $^{**}P < 0.01$, $^{***}P < 0.001$, $^{****}P < 0.0001$, ns = not significant.
