# Supplementary material for: Elimination of senescent cells by β-galactosidase-targeted prodrug attenuates inflammation and restores physical function in aged mice
Source: Cell Res. 2020 Apr 27;30(7):574–89. doi: 10.1038/s41422-020-0314-9 (PMC7184167; doi:10.1038/s41422-020-0314-9)
Supplement: Supplementary file 2 — Supplementary information Figure S2 [file 41422_2020_314_MOESM2_ESM.pdf]

Supplementary information, Figure S2

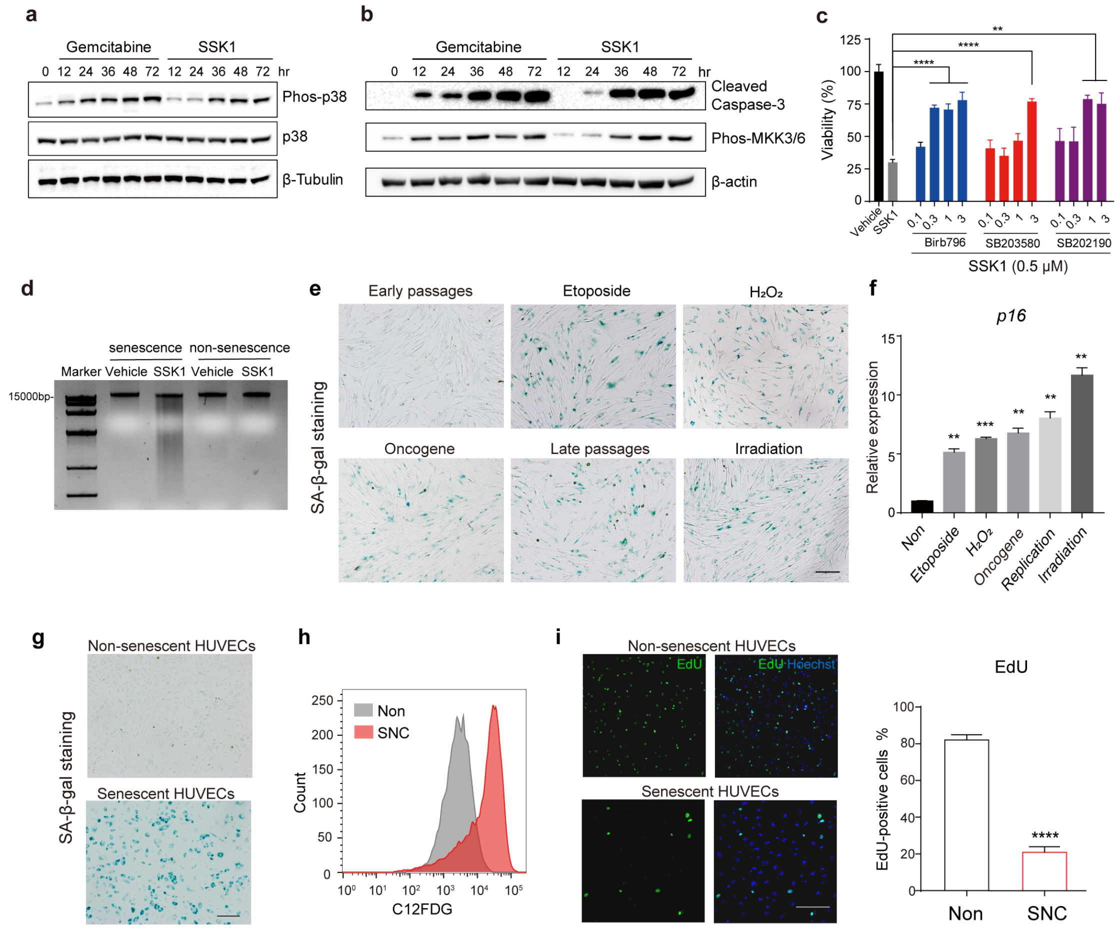

Supplementary information Fig. S2: The mechanism of SSK1 kill senescent cells and identification of cellular senescence.

a, **b** Western blot detection of phos-p38 MAPK (**a**), phos-MKK3/6 and cleaved caspase 3 (**b**) in senescent NBFs after treatment with gemcitabine (0.05 μM) or SSK1 (0.5 μM) for 3 days. **c** Quantitation of cell viability of senescent NBFs treated with vehicle (DMSO), SSK1 (0.5 μM), or the combination of SSK1 and p38 inhibitors for 3 days ($n = 4$). **d** Detection of the mitochondrial DNA extracted from senescent and non-senescent cells treated with vehicle or SSK1 (0.5 μM) for 3 days. **e**, **f** Identification of non-senescent and senescent HEFs. Representative images of SA-β-gal staining (**e**) and *p16* expression (**f**) of non-senescent and senescent HEFs induced by etoposide, H_2O_2 (200 μM), oncogene (*Kras*^{G12V}), replication (﹥25 passages), or irradiation (10 Gy) after

SSK1 treatment. Scale bars, 200 μm. **g**-**i** Identification of non-senescent and senescent HUVECs. (**g**) Representative images of SA-β-gal staining (X-gal) of non-senescent and senescent HUVECs. (**h**) Representative flow cytometric histogram detecting SA-β-gal activity using C12FDG in non-senescent (Non) and senescent (SNC) HUVECs. (**i**) Representative images (**left**) and quantification (**right**) of EdU-positive cells in non-senescent and senescent HUVECs. DNA (blue) was staining with Hoechst 33342. Green cells show EdU-positive cells ($n = 3$). Scale bars, 200 μm. Data are presented as means ± SEM. '*n*' represents number of biological replicates. Unpaired two-tailed *t*-test for (**i**), one-way ANOVA test for (**c**) and (**f**), $^{**}P < 0.01$, $^{***}P < 0.001$, $^{****}P < 0.0001$.
