# Supplementary material for: Elimination of senescent cells by β-galactosidase-targeted prodrug attenuates inflammation and restores physical function in aged mice
Source: Cell Res. 2020 Apr 27;30(7):574–89. doi: 10.1038/s41422-020-0314-9 (PMC7184167; doi:10.1038/s41422-020-0314-9)
Supplement: Supplementary file 3 — Supplementary information Figure S3 [file 41422_2020_314_MOESM3_ESM.pdf]

Supplementary information, Figure S3

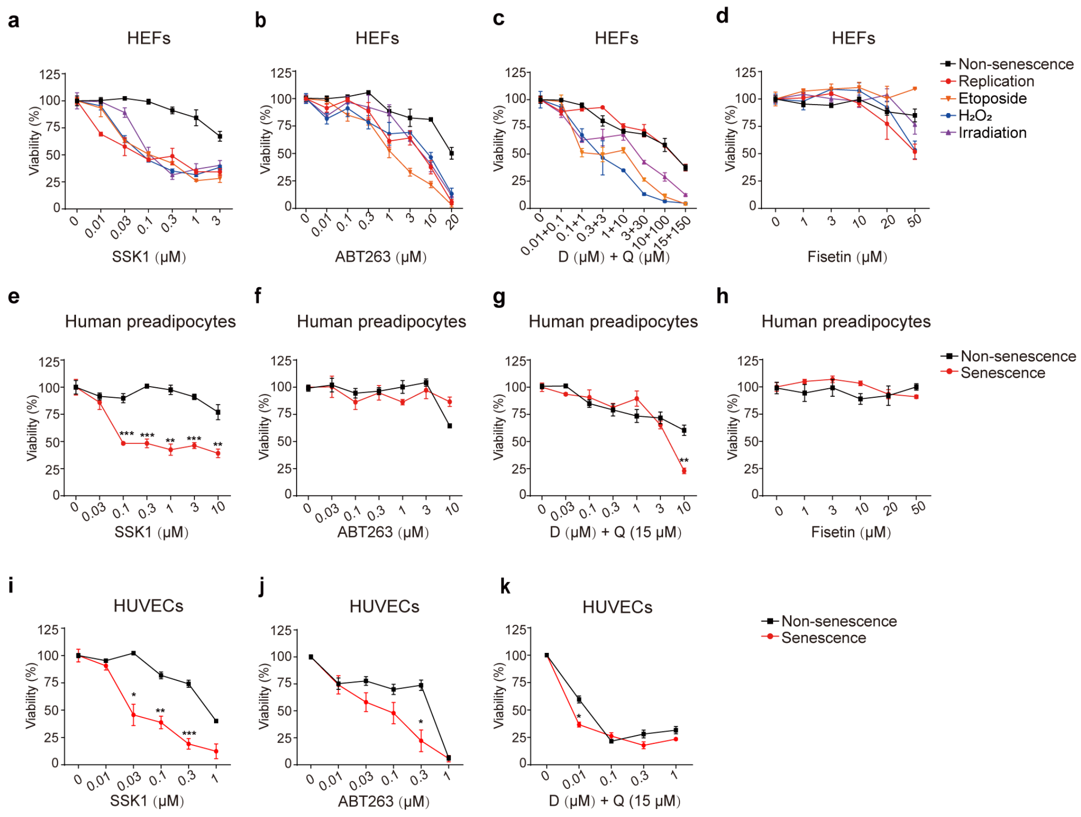

Supplementary information Fig. S3: Compare SSK1 with reported senolytics in vitro.

a-d Quantification of cell viability of non-senescent and replication, etoposide, H_2O_2 (200 μM) and irradiation (10 Gy) -induced senescent HEFs incubated with the indicated concentrations of SSK1 (**a**), ABT263 (**b**), dasatinib plus quercetin (D + Q) (**c**) or fisetin (**d**) for 3 days (*n* = 3). Significance analysis is shown in Supplementary information Table S2. **e-h**, Quantification of cell viability of non-senescent and replication-induced human preadipocytes incubated with the indicated concentrations of SSK1 (**e**), ABT263 (**f**), D + Q (**g**) or fisetin (**h**) for 3 days (*n* = 3). **i-k**, Quantification of cell viability of non-senescent and senescent HUVECs incubated with the indicated concentrations of SSK1 (**i**), ABT263 (**j**), D + Q (**k**) for 3 days (*n* = 3). Cell numbers were quantified using Hoechst 33342 staining and dead cells were exclude by PI staining, and then cell viability was plotted. Data are presented as means ± SEM. '*n*' represents number of biological replicates. Two-way ANOVA test for (**a**)-(**d**), unpaired two-tailed *t*-test

for (**e**)-(**k**), $*P < 0.05$, $**P < 0.01$, $***P < 0.001$, $****P < 0.0001$.
