# Supplementary material for: Elimination of senescent cells by β-galactosidase-targeted prodrug attenuates inflammation and restores physical function in aged mice
Source: Cell Res. 2020 Apr 27;30(7):574–89. doi: 10.1038/s41422-020-0314-9 (PMC7184167; doi:10.1038/s41422-020-0314-9)
Supplement: Supplementary file 4 — Supplementary information Figure S4 [file 41422_2020_314_MOESM4_ESM.pdf]

Supplementary information, Figure S4

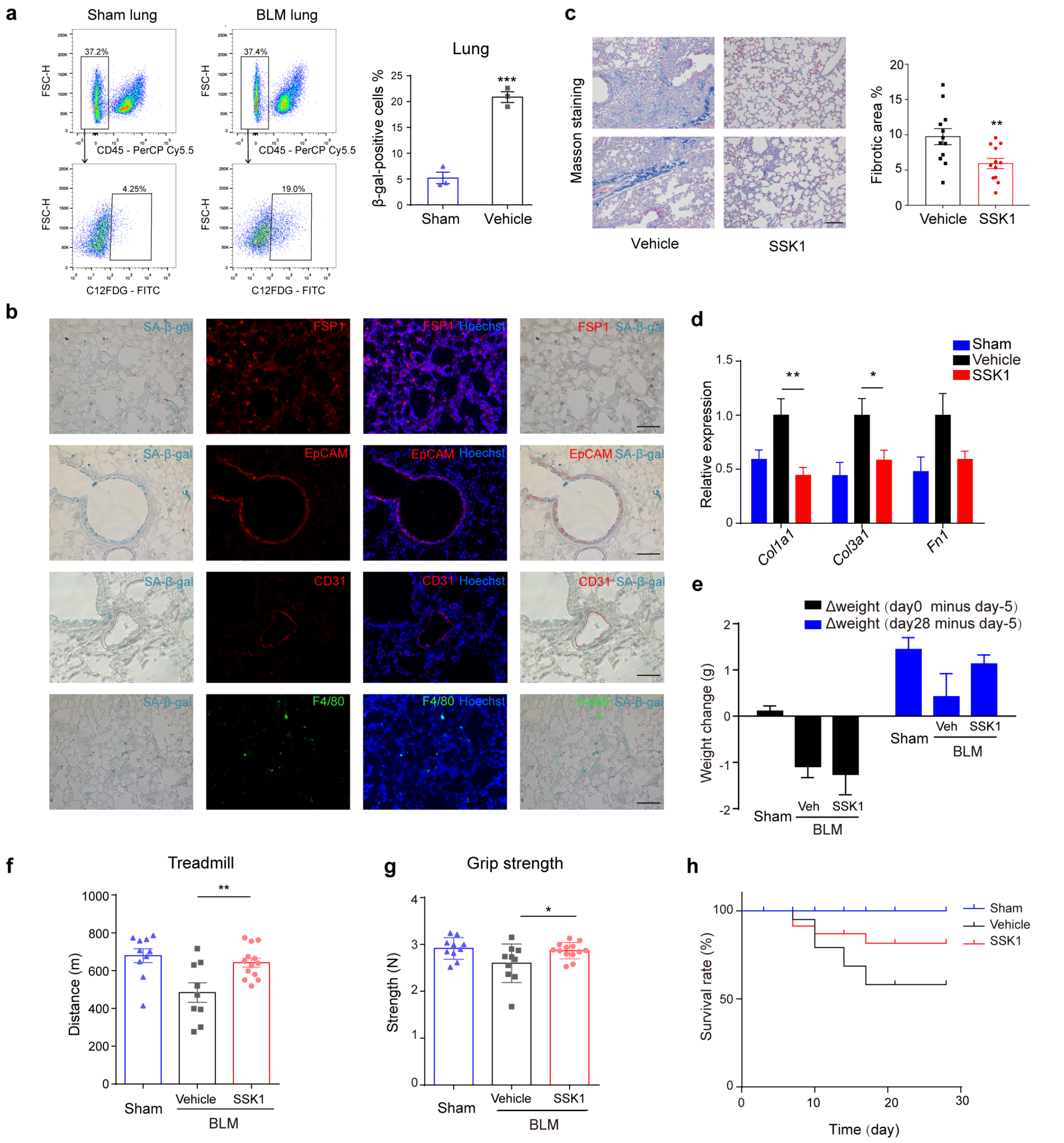

Supplementary information Fig. S4: SSK1 attenuates lung fibrosis and improves physical function of bleomycin-induced mice.

a Flow cytometry analysis of lung cell suspensions from sham surgery (Sham) and bleomycin-induced mice. The representative dot plots of the non-hematopoietic cells ($CD45^-$) and the β-gal-positive populations by staining C12FDG (**left**); The Percentage of non-hematopoietic β-gal-positive cells (**right**) (*n* = 3). **b** Representative images of co-immunofluorescence staining of FSP1 (S100A4), EpCAM, CD31 and F4/80 with SA-β-gal staining in bleomycin-

injured lungs, Scale bars, 200 μm. **c** Representative Masson fibrosis staining (**left**) and quantification of the proportion of fibrosis (**right**) of lung paraffin sections from bleomycin-injured mice after vehicle or SSK1 (0.5 mg/kg) treatment (Vehicle-treated, n = 12; SSK1-treated, n = 12). Scale bar, 200 μm. **d** Expression of fibrosis associated genes by RT-qPCR in lung tissues of sham surgery mice (Sham) and bleomycin-injured mice treated with vehicle or SSK1 (Sham, n = 9; vehicle-treated, n = 9; SSK1-treated, n = 10). **e** Body weight change of sham surgery and bleomycin-injured mice (BLM) 5 days after surgery and 4 weeks of vehicle or SSK1 treatment (Sham, n = 10; vehicle-treated, n = 9; SSK1-treated, n = 12). **f** Exhaustion distance in treadmill in sham surgery and bleomycin-injured mice treated with vehicle or SSK1 (Sham, n = 10; vehicle-treated, n = 9; SSK1-treated, n = 13). **g** Grip strength of four limbs in sham surgery and bleomycin-injured mice treated with vehicle or SSK1 (Sham, n = 10; vehicle-treated, n = 10; SSK1-treated, n = 13). **h** Percentage of survival of sham surgery and bleomycin-injured mice treated with vehicle or SSK1 (Sham, n = 10; vehicle-treated, n = 19; SSK1-treated, n = 19). Each data point represents an individual mouse. '*n*' represents number of mice. Data are presented as means ± SEM. Unpaired two-tailed *t*-test, $^*P < 0.05$, $^{**}P < 0.01$.
