# Supplementary material for: Elimination of senescent cells by β-galactosidase-targeted prodrug attenuates inflammation and restores physical function in aged mice
Source: Cell Res. 2020 Apr 27;30(7):574–89. doi: 10.1038/s41422-020-0314-9 (PMC7184167; doi:10.1038/s41422-020-0314-9)
Supplement: Supplementary file 5 — Supplementary information Figure S5 [file 41422_2020_314_MOESM5_ESM.pdf]

Supplementary information, Figure S5

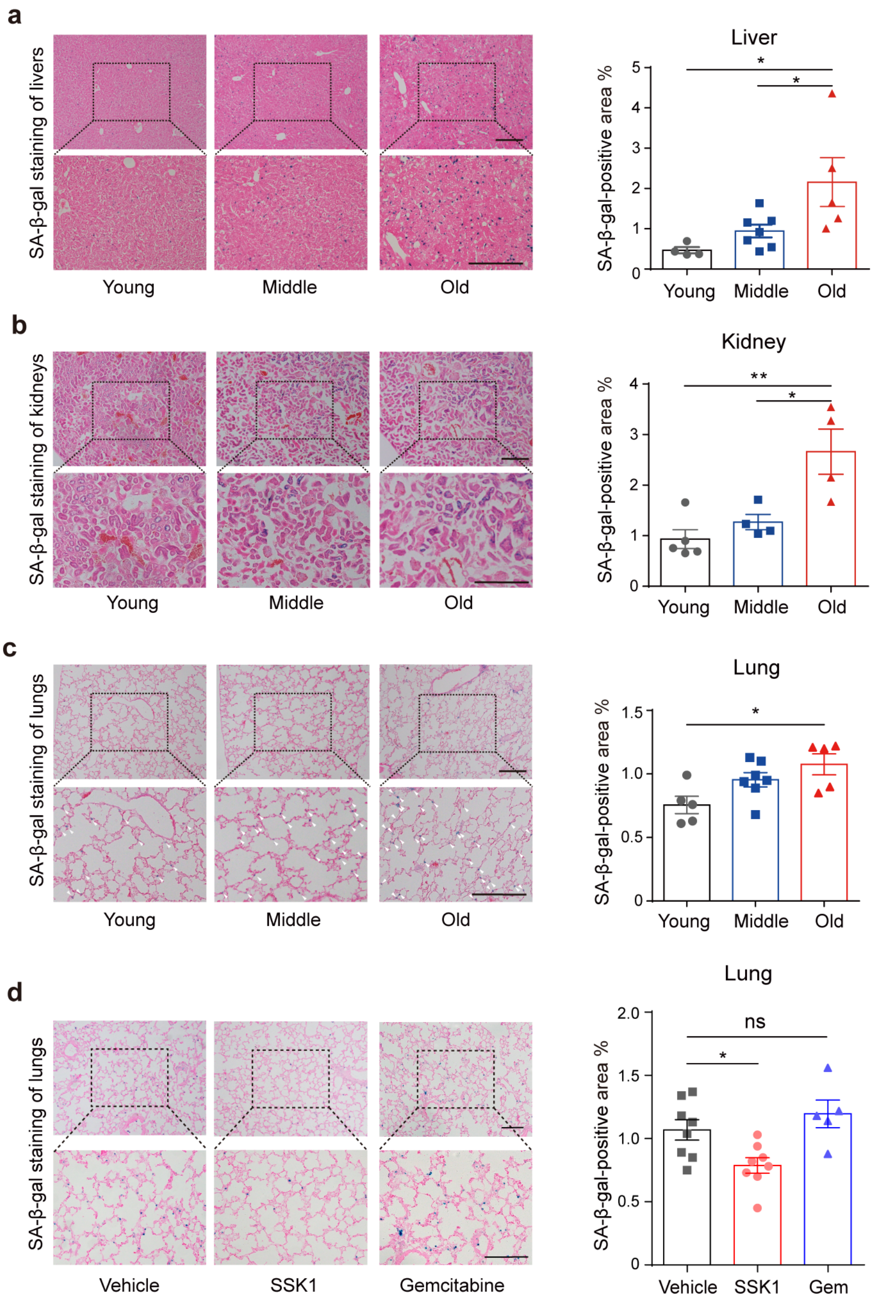

Supplementary information Fig. S5: SA-β-gal-positive cells increase in kidneys, livers and lungs of aged mice and decrease in lungs treated with SSK1.

a Representative images (**left**) and quantification (**right**) of SA-β-gal staining of livers of young, middle-aged, and old mice (n = 4, 7, 5 for each group respectively). **b** Representative images (**left**) and quantification (**right**) of SA-β-gal staining of kidneys of young, middle-aged, and old mice (n = 5, 4, 4 for

each group respectively). **c** Representative images (**left**) and quantification (**right**) of SA-β-gal staining of lungs of young, middle-aged, and old mice (*n* = 5, 7, 5 for each group respectively). **d** Representative images (**left**) and quantification (**right**) of SA-β-gal staining of lungs of old mice after vehicle, SSK1 (0.5 mg/kg) or gemcitabine (0.5 mg/kg) treatment for 8 weeks (vehicle- and SSK1-treated, *n* = 8; gemcitabine-treatment, *n* = 5). Scale bars, 200 μm. Each data point represents an individual mouse. '*n'* represents number of mice. Data are presented as means ± SEM. Unpaired two-tailed *t*-test, $^{*}P < 0.05$, $^{**}P < 0.01$, ns = not significant.
