# Supplementary material for: Elimination of senescent cells by β-galactosidase-targeted prodrug attenuates inflammation and restores physical function in aged mice
Source: Cell Res. 2020 Apr 27;30(7):574–89. doi: 10.1038/s41422-020-0314-9 (PMC7184167; doi:10.1038/s41422-020-0314-9)
Supplement: Supplementary file 6 — Supplementary information Figure S6 [file 41422_2020_314_MOESM6_ESM.pdf]

Supplementary information, Figure S6

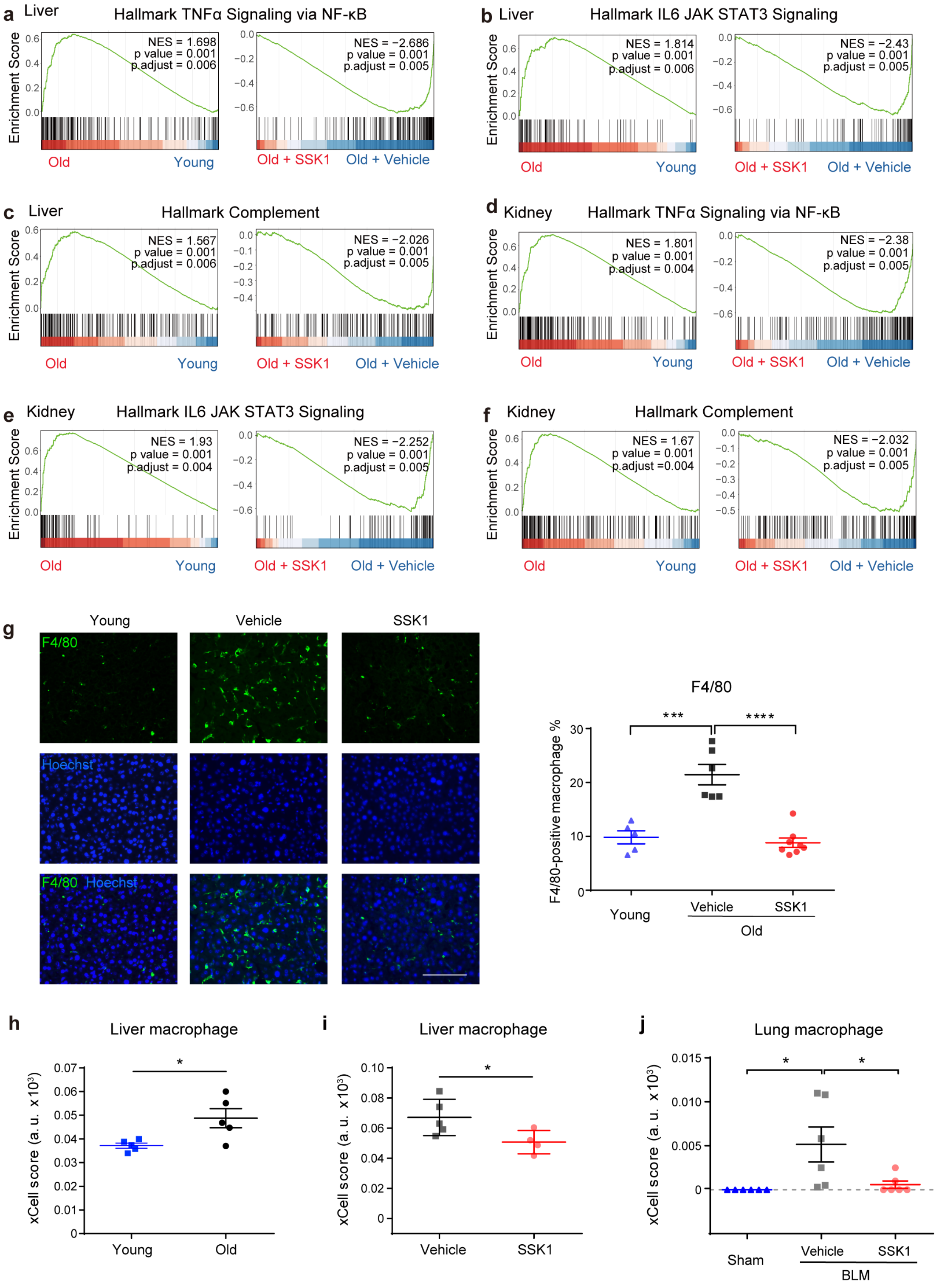

Supplementary information Fig. S6: SSK1 attenuates the inflammation associated signatures and declines macrophages.

a-c GSEA of statistically significant gene sets in livers: Hallmark TNFα signaling

via NF-κB (**a**), Hallmark IL6 JAK STAT3 signaling (**b**) and Hallmark Complement (**c**) enriched in old mice compared with young mice, and down-regulated in the old mice treated with SSK1 compared with vehicle (vehicle-treated, n = 5; SSK1-treated, n = 4). **d**-**f** GSEA of statistically significant gene sets in kidneys: Hallmark TNFα signaling via NF-κB (**d**), Hallmark IL6 JAK STAT3 signaling (**e**) and Hallmark Complement (**f**) enriched in old mice compared with young mice, and down-regulated in the old mice treated with SSK1 compared with vehicle (vehicle-treated, n = 5; SSK1-treated, n = 5). **g** Representative images (**left**) and quantification (**right**) of liver F4/80 staining of young mice and aged mice after vehicle or SSK1 (0.5 mg/kg) treatment (young, n = 5; Vehicle-treated, n = 6; SSK1-treated, n = 8). Scale bar, 200 μm. **h** xCell analysis of RNA-seq data to predict changes in macrophage infiltration in the livers of young (4 month) and aged mice (19 month) (young, n = 5; aged, n = 5). **i** xCell analysis of RNA-seq data to predict changes in macrophage infiltration of old mice after treated with SSK1 (Vehicle-treated, n = 5; SSK1-treated, n = 4). **j** xCell analysis of RNA-seq data to predict changes in macrophage infiltration in the lungs of sham surgery (Sham) and bleomycin- induced injury lungs treated with vehicle or SSK1 (0.5 mg/kg) (Sham, n = 6; vehicle-treated, n = 6; SSK1-treated, n = 6). Each data point represents an individual mouse. '*n*' represents number of mice. Data are presented as means ± SEM. Unpaired two-tailed *t*-test, $^{*}P < 0.05$, $^{***}P < 0.001$, $^{****}P < 0.0001$.
