# Supplementary material for: Elimination of senescent cells by β-galactosidase-targeted prodrug attenuates inflammation and restores physical function in aged mice
Source: Cell Res. 2020 Apr 27;30(7):574–89. doi: 10.1038/s41422-020-0314-9 (PMC7184167; doi:10.1038/s41422-020-0314-9)
Supplement: Supplementary file 7 — Supplementary information Figure S7 [file 41422_2020_314_MOESM7_ESM.pdf]

Supplementary information, Figure S7

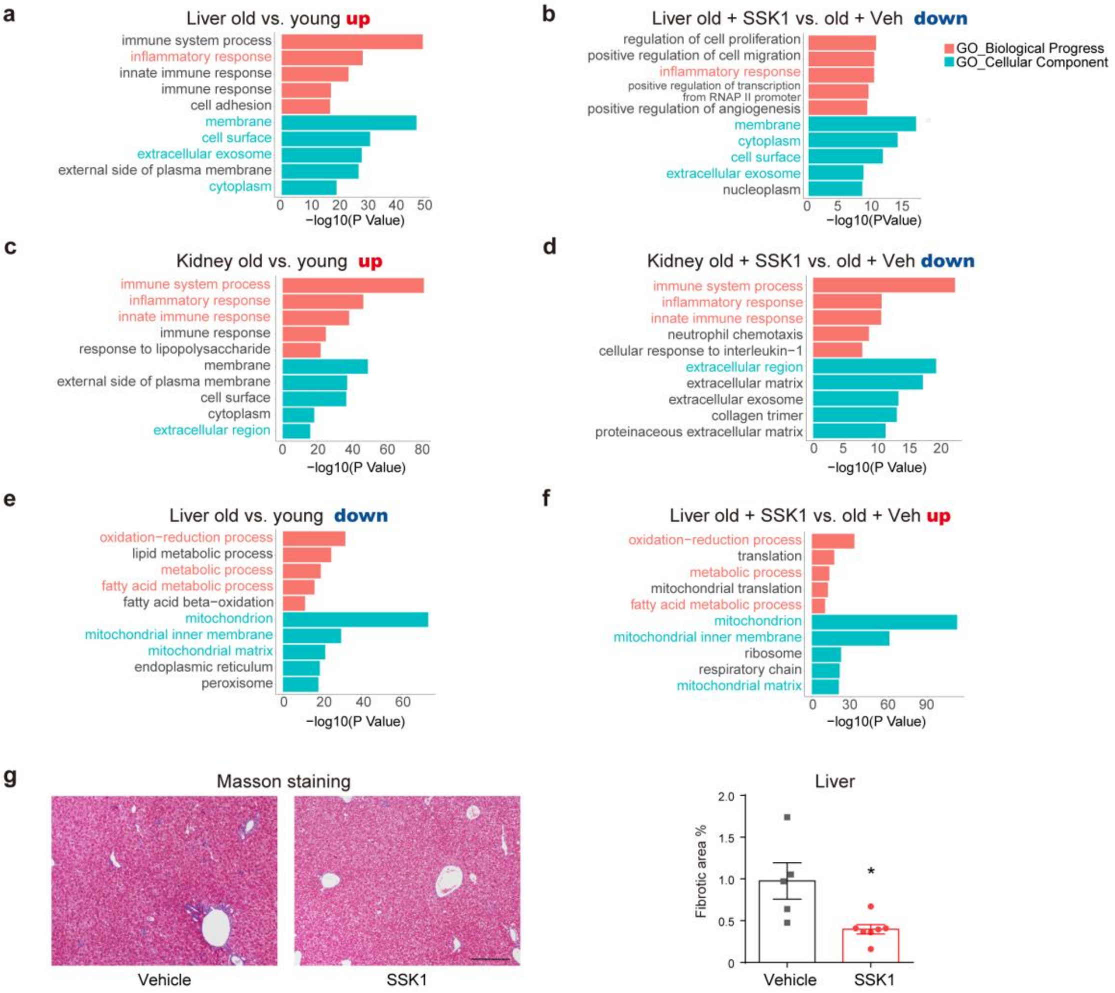

Supplementary information Fig. 7: Gene ontology analysis of SSK1 treatment.

a-d GO Terms of up-regulated genes in the livers (**a**) and kidneys (**c**) of old mice compared with young mice; And GO terms for down-regulated genes in the livers (**b**) and kidneys (**d**) of SSK1-treated old mice relative to vehicle-treated old mice. **e**, **f** GO Terms of down-regulated genes in the livers of old mice compared with young mice (**e**) and GO terms for up-regulated genes of SSK1-treated old mice relative to vehicle-treated old mice (**f**). Terms both in **a** and **b** (**c** and **d; e** and **f**) are in red or cyan. Details of GO analysis shown in Table S3. **g** Representative Masson fibrosis staining (**left**) and quantification of the proportion of fibrosis (**right**) of liver paraffin sections from old mice treated with vehicle or SSK1 (Vehicle-treated, *n* = 5; SSK1-treated, *n* = 7). Scale bar, 200

μm. Each data point represents an individual mouse. '*n*' represents number of mice. Data are presented as means ± SEM. Unpaired two-tailed *t*-test, $*P < 0.05$.
