# Supplementary material for: Elimination of senescent cells by β-galactosidase-targeted prodrug attenuates inflammation and restores physical function in aged mice
Source: Cell Res. 2020 Apr 27;30(7):574–89. doi: 10.1038/s41422-020-0314-9 (PMC7184167; doi:10.1038/s41422-020-0314-9)
Supplement: Supplementary file 8 — Supplementary information Figure S8 [file 41422_2020_314_MOESM8_ESM.pdf]

Supplementary information, Figure S8

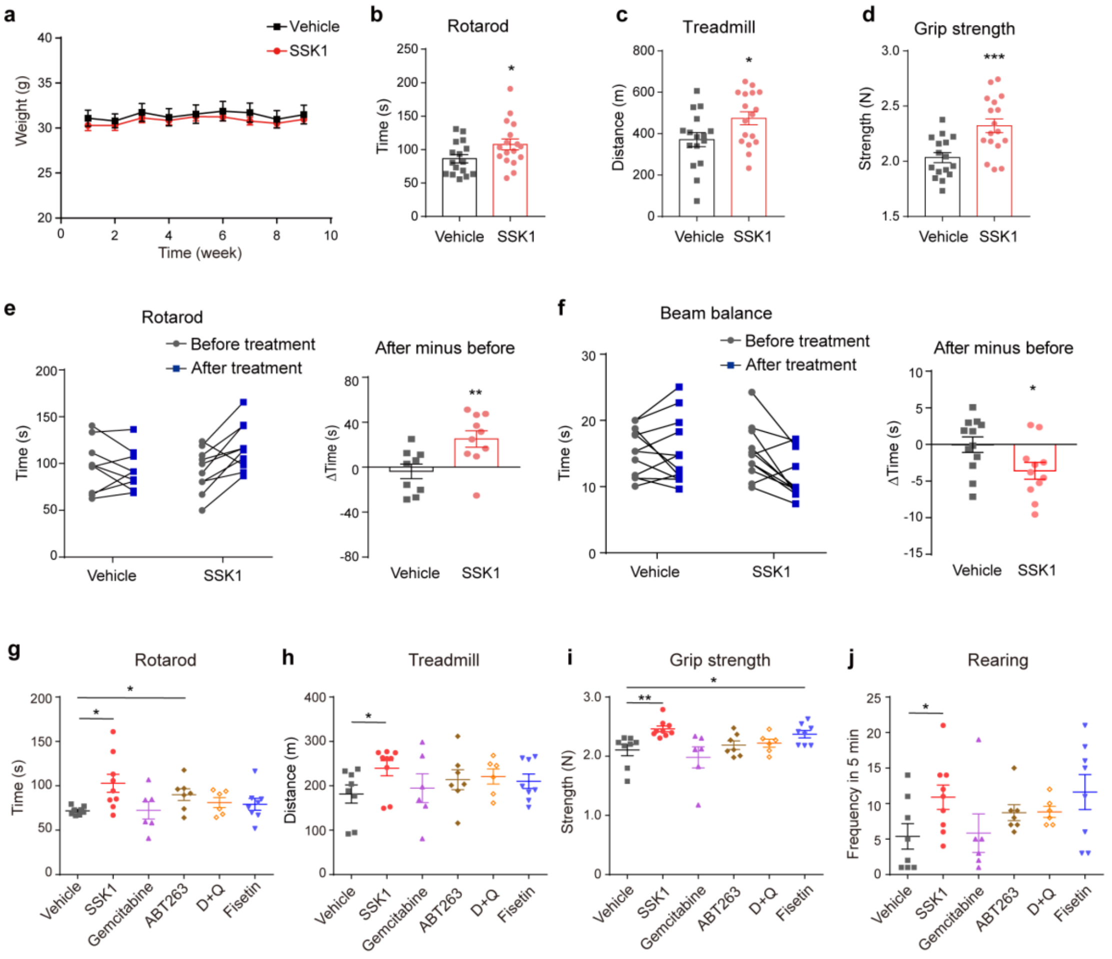

Supplementary information Fig. 8: SSK1 improves physical function.

a Body weight of vehicle or SSK1 treated old female mice within the experimental period. **b-d** Quantification of maximal rotarod time (**b**), treadmill distance (**c**), grip strength (**d**) for old male mice treated with vehicle (Veh) or SSK1 (Vehicle-treated, *n* = 16; SSK1-treated, *n* = 17). **e** Quantification of maximal rotarod time for old mice before and after treatment with vehicle or SSK1 (**left**) and the maximal rotarod time after treatment minus the maximal time of rotarod before treatment with vehicle or SSK1 (**right**) (vehicle-treated, *n* = 9, SSK1-treated, *n* = 10). **f** Quantification of the time to cross the balance beam for old mice before and after treatment with vehicle or SSK1 (**left**) and the time to cross the balance beam minus the time before treatment with vehicle or SSK1 (**right**) (vehicle-treated, *n* = 12, SSK1-treated, *n* = 11). **g-j**

Quantification of maximal time in rotarod (**g**), the exhaustion distance in treadmill (**h**), grip strength (**i**), and the number of rearing exploration times in 5 min (**j**) for old mice treated with vehicle or SSK1 compared with gemcitabine, ABT263, D + Q, and fisetin (*n* = 8, 9, 6, 7, 6, 8 for each group respectively). Old mice (20-22-month-old) were intraperitoneally injected with SSK1 (0.5 mg/kg), gemcitabine (0.5 mg/kg), ABT263 (2 mg/kg), dasatinib (1 mg/kg) plus quercetin (10 mg/kg), fisetin (20 mg/kg) or vehicle (DMSO) for continued 3 days every 2 weeks for 8 weeks. Each data point represents an individual mouse. '*n*' represents number of mice. Data are presented as means ± SEM. Unpaired two-tailed *t*-test, *$P < 0.05$, **$P < 0.01$, ***$P < 0.001$.
