# Supplementary material for: Elimination of senescent cells by β-galactosidase-targeted prodrug attenuates inflammation and restores physical function in aged mice
Source: Cell Res. 2020 Apr 27;30(7):574–89. doi: 10.1038/s41422-020-0314-9 (PMC7184167; doi:10.1038/s41422-020-0314-9)
Supplement: Supplementary file 9 — Supplementary information Figure S9 [file 41422_2020_314_MOESM9_ESM.pdf]

Supplementary information, Figure S9

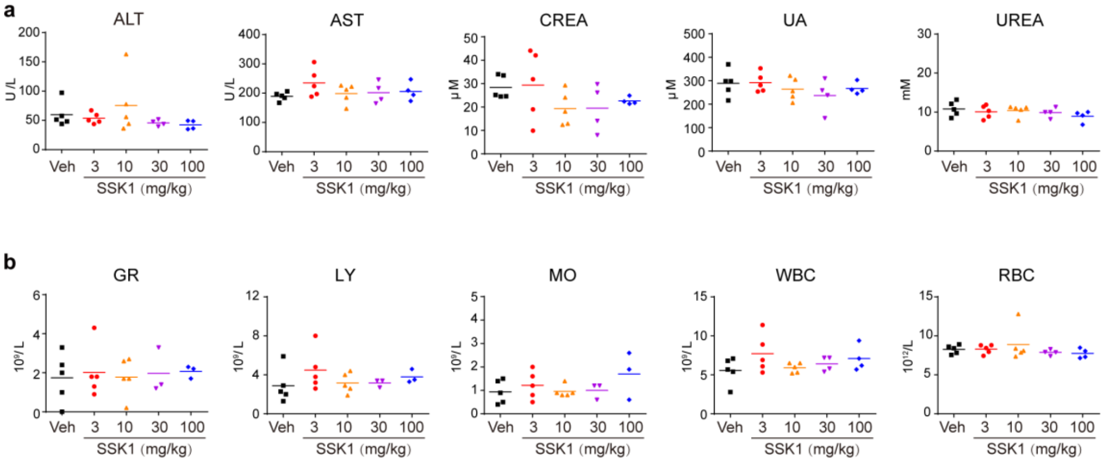

Supplementary information Fig. 9: Serum biochemical test and routine analysis of old mice treated with high dosage of SSK1.

a Serum biochemical test of mice treated with increasing doses of SSK1. The level of Alanine transaminase (ALT), aspartate transaminase (AST), creatinine (CREA), uric acid (UA) and carbamide (UREA) in old mice after vehicle and SSK1 (3, 10, 30, 100 mg/kg) treatment for 5 weeks with 3 injections a week (n = 5, 5, 5, 4, 4 for each group respectively). **b** Routine analysis of blood of mice treated with increasing doses of SSK1. The number of granulocytes (GR), lymphocytes (LY), monocytes (MO), white blood cells (WBC) and red blood cells (RBC) of old mice after vehicle and SSK1 (3, 10, 30, 100 mg/kg) treatment for 5 weeks with 3 injections a week (For GR, LY and MO: n = 5, 5, 5, 3, 3 for each group respectively; For WBC and RBC: n = 5, 5, 5, 4, 4 for each group respectively). All Data are presented as means ± SEM. Each data point represents an individual mouse. '*n'* represents the number of mice. Statistical significance was calculated using one-way ANOVA test, all the data were not significant.
