# Supplementary material for: Elimination of senescent cells by β-galactosidase-targeted prodrug attenuates inflammation and restores physical function in aged mice
Source: Cell Res. 2020 Apr 27;30(7):574–89. doi: 10.1038/s41422-020-0314-9 (PMC7184167; doi:10.1038/s41422-020-0314-9)
Supplement: Supplementary file 10 — Supplementary information Figure S10 [file 41422_2020_314_MOESM10_ESM.pdf]

Supplementary information, Figure S10

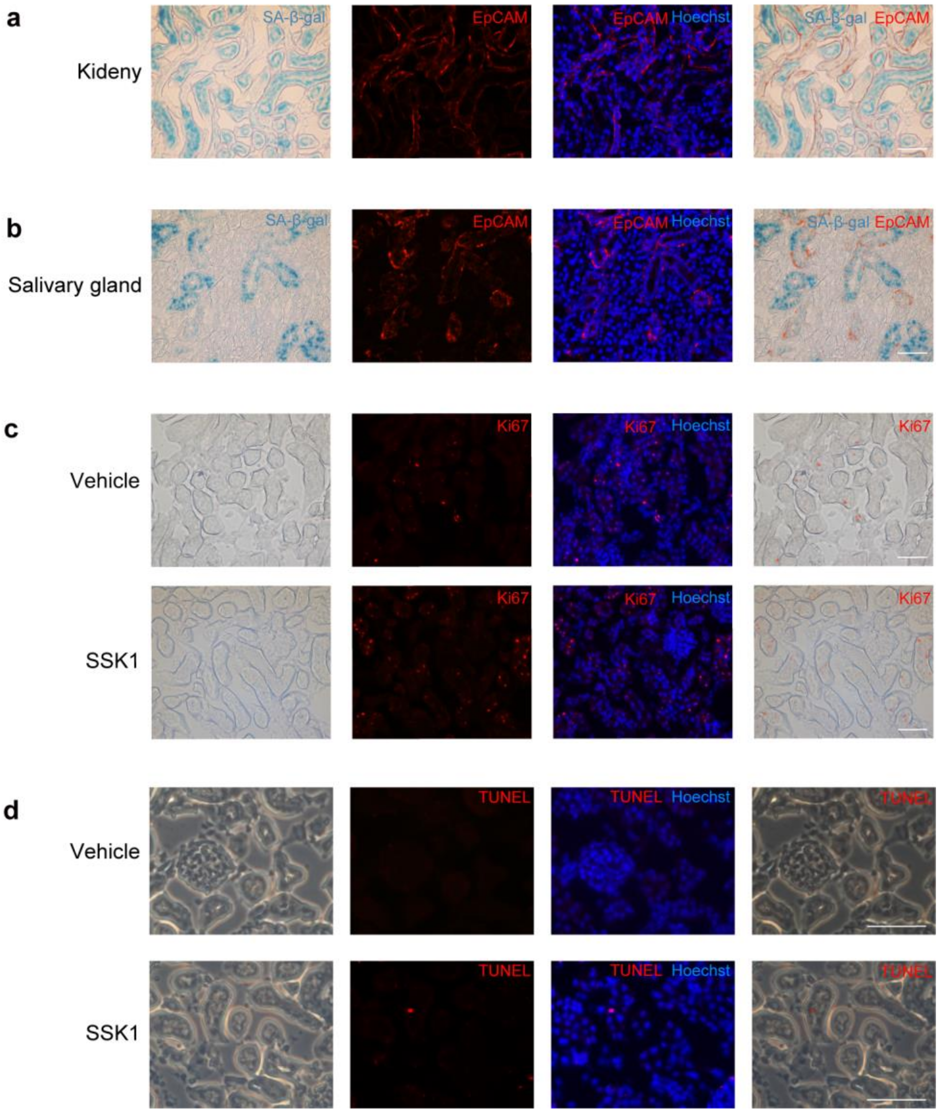

Supplementary information Fig. 10: Endogenous acid β-gal staining of kidneys and salivary glands and the apoptosis and proliferation detection after SSK1 treatment.

a, **b** Representative images of co-immunofluorescence staining of EpCAM with endogenous acid β-gal staining in kidneys **(a)** and salivary glands **(b)**. Scale bars, 200 μm. **c** Representative images of Ki67 staining of kidneys after vehicle or SSK1 treatment. Scale bars, 200 μm. **d** Representative images of TUNEL apoptosis staining of kidneys after vehicle or SSK1 treatment. Scale bars, 200 μm.
