# Supplementary material for: Elimination of senescent cells by β-galactosidase-targeted prodrug attenuates inflammation and restores physical function in aged mice
Source: Cell Res. 2020 Apr 27;30(7):574–89. doi: 10.1038/s41422-020-0314-9 (PMC7184167; doi:10.1038/s41422-020-0314-9)
Supplement: Supplementary file 11 — Supplementary information Figure S11 [file 41422_2020_314_MOESM11_ESM.pdf]

Supplementary information, Figure S11

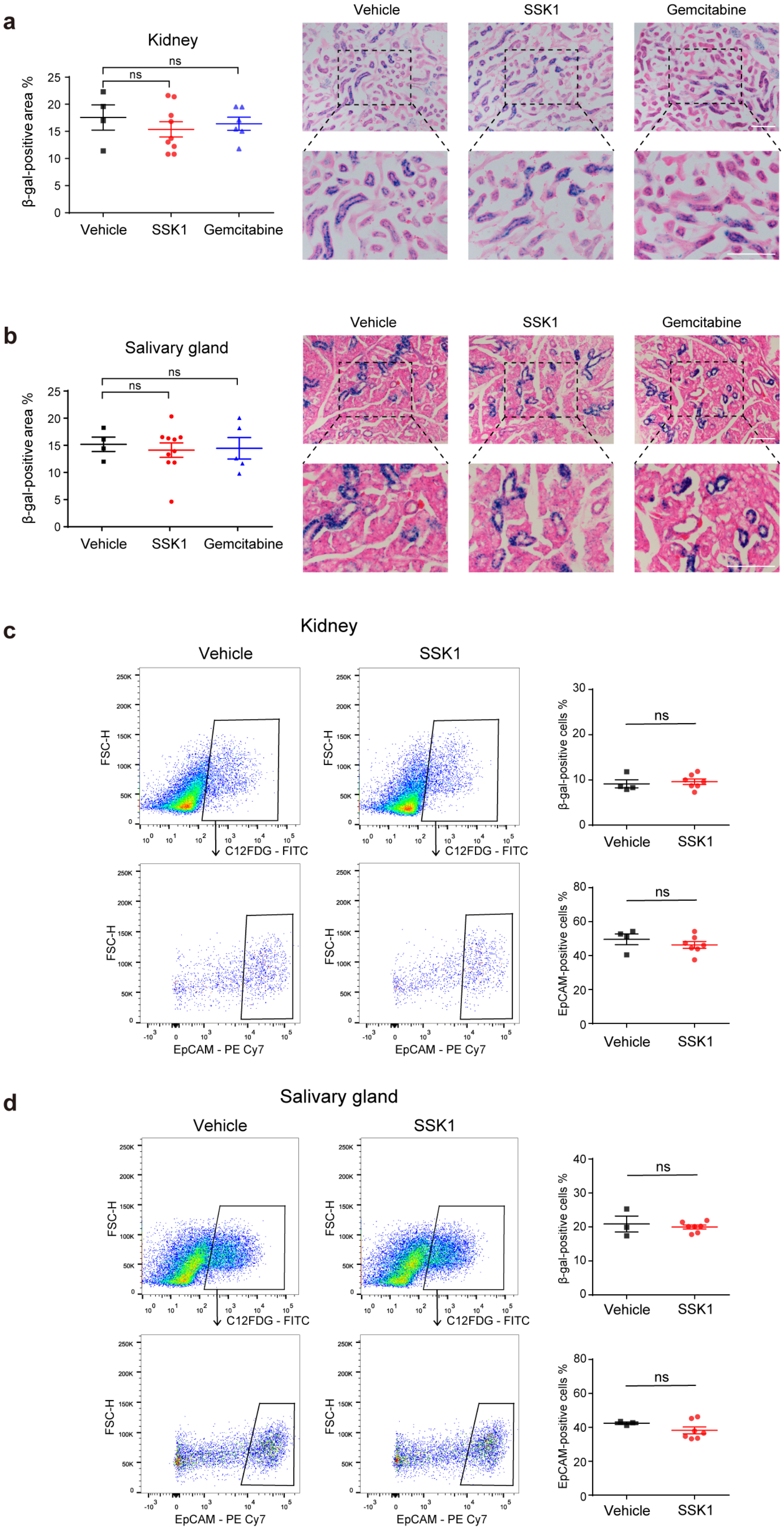

Supplementary information Fig. S11: Quantification of endogenous acid β-gal change in kidneys and salivary glands after treated with SSK1.

a Representative images (**right**) and quantification (**left**) of β-gal staining of kidneys after vehicle, SSK1 or gemcitabine treatment (vehicle-treated, *n* = 4; SSK1-treated, *n* = 9; gemcitabine-treated, *n* = 6). Scale bars, 100 μm. **b** Quantification (**left**) and representative images (**right**) and of β-gal staining of submandibular glands after vehicle, SSK1 or gemcitabine treatment (vehicle-treated, *n* = 4; SSK1-treated, *n* = 10; gemcitabine-treated, *n* = 5). Scale bars, 100 μm. **c**, **d** Flow cytometry analysis of kidney **(c)** and salivary gland **(d)** cell suspensions from vehicle or SSK1 treated mice. Representative dot plots of endogenous acid β-gal-positive populations by staining C12FDG (without chloroquine reagent) and the major β-gal-positive cell type ($EPCAM^+$ epithelial cells) (**left**); The percentages of β-gal-positive cells and the proportions of $EPCAM^+$ epithelial cells in β-gal-positive cells (**right**) (Vehicle-treated, *n* = 4 for kidney and *n* = 3 for salivary gland; SSK1-treated, *n* = 7 for both kidney and salivary gland). Each data point represents an individual mouse. '*n'* represents number of mice. Unpaired two-tailed *t*-test, ns = not significant.
